# Assessment of interocular symmetry of choroidal vascularity index and thickness in patients with systemic sclerosis: a prospective study

**DOI:** 10.3389/fmed.2024.1513679

**Published:** 2025-01-16

**Authors:** Anna Raciborska, Barbara Pieklarz, Ewa Gińdzieńska-Sieśkiewicz, Agnieszka Zonenberg, Otylia Kowal-Bielecka, Joanna Konopińska, Diana A. Dmuchowska

**Affiliations:** ^1^Ophthalmology Department, Medical University of Bialystok, Bialystok, Poland; ^2^Department of Rheumatology and Internal Diseases, Medical University of Bialystok, Bialystok, Poland

**Keywords:** choroidal vascularity index, choroidal thickness, symmetry, systemic sclerosis, interocular comparison

## Abstract

**Purpose:**

Systemic sclerosis (SSc) affects blood vessels, internal organs, and skin. In ophthalmology, SSc impacts the choroid. The choroidal vascularity index (CVI) measures the vascular component of the choroid and may serve as a biomarker for the disease staging and prognosis. Studies have reported reduced choroidal thickness and altered CVI in SSc, which supports the theory of vascular damage. This study aimed to examine interocular symmetry in choroidal parameters among SSc patients. It has provided the insight into the disease symmetry and assessed the representativeness of examining one eye.

**Methods:**

This prospective single-center cross-sectional study included 33 patients with SSc and 40 healthy controls. The patients underwent ophthalmological examination (including refraction, visual acuity, IOP, biometry, slit-lamp biomicroscopy, dilated fundus examination, and spectral-domain optical coherence tomography) and rheumatological evaluation. Various parameters of the choroid in the macular and peripapillary regions were analyzed, including choroidal thickness, choroidal volume, and CVI. The interocular asymmetry in the choroidal parameters was quantified using signed and absolute differences. The correlation analysis between the left and right eyes was based on the intraclass correlation coefficient (ICC), Spearman’s correlation coefficient, and paired Wilcoxon test.

**Results:**

There were no significant differences in the macular and peripapillary choroidal parameters between fellow eyes in both SSc patients and controls (*p* > 0.05). The parameter that showed the lowest correlation among those examined was CVI—in both groups, as well as in both examined areas. The interocular correlation of choroidal parameters was stronger in the peripapillary area than in the macular area in both groups. In general, the results were confirmed in subgroup analyses stratified according to sex, SSc subtype, Scl70 antibody positivity and previous and/or active digital ulcers.

**Conclusion:**

There is interocular symmetry of the choroidal parameters in patients with SSc and controls included in our study. The parameters from one eye are representative of the fellow eye of a given patient. This conclusion may contribute to the design and interpretation of future studies. It also broadens our knowledge of SSc pathophysiology.

## Introduction

Systemic sclerosis (SSc) is a chronic autoimmune disease characterized by vasculopathy and tissue fibrosis (1). The disease’s etiology is unknown, but the immune system activation plays a key role in its progression. This disease causes skin fibrosis and contributes to the damage in internal organs and blood vessels (2). From an ophthalmological point of view, SSc may affect the ocular adnexa, anterior and posterior segments of the eye, orbit, and extraocular muscles (3–7). Patients with systemic sclerosis (SSc) are at an increased risk of developing glaucomatous optic neuropathy (8–10).

Choroidal involvement has also been described previously. The choroid is responsible for supplying oxygen to the outer retina and removing metabolic products (11). Its visualization is possible owing to optical coherence tomography (OCT). The parameters characterizing the choroid are: choroidal thickness (CT), volume (CV), and choroidal vascularity index (CVI). CT is a rather variable parameter that depends on axial length (AXL), age, intraocular pressure (IOP), sex, and blood pressure (12). The CVI, the ratio of the choroidal luminal area (LA) to total choroidal area (TCA), is a reliable measure of both vascular and stromal choroidal components (12). Though not yet in routine clinical use, the CVI may become a noninvasive biomarker for diagnosing, assessing severity, and tracking progression of systemic diseases, particularly those with vascular involvement, such as diabetes (13–15), systemic lupus erythematosus, systemic sclerosis, and Behçet’s disease (16). Ataş et al. showed that the more severe the stage of SSc, the lower the CVI index, which is valuable information for monitoring the disease progression (17).

In the case of patients with SSc, in the macula, we were able to show a lower choroidal thickness and volume as well as a higher CVI, which indicates that stromal involvement appears to dominate the vascular component (18). We also investigated peripapillary choroidal parameters (19). No significant differences were found between the patients with SSc and controls, except for the peripapillary CVI that was significantly lower in the patients with SSc. We suggest that this is probably due to a decrease in the vascular layer, which would partially explain the increased risk of glaucoma in patients with SSc. Due to the end-arterial nature of the choroidal vasculature and the existence of watershed zones in the choroid (20), we analyzed the macular and peripapillary regions of the choroid separately.

Choroidal involvement has been investigated in many diseases but without interocular comparisons (21, 22). In contrast, the symmetry of choroidal parameters in healthy patients has been widely described in the related literature (23–28). However, the data on choroidal symmetry in patients with ocular or systemic diseases are scarce. Given the growing interest in CVI as a superior marker to choroidal thickness for diagnosis, prognosis, and treatment, this gap in the literature prompted our study. Recently, we have shown that there is some asymmetry in choroidal parameters in patients with diabetic retinopathy, depending on the presence of concomitant diabetic macular edema (13).

To the best of our knowledge, so far, no studies have assessed the symmetry of choroidal parameters in the eyes of patients with systemic sclerosis. It is the fact that deepens our insight into the pathophysiology of SSc. It may also facilitate the search for ocular biomarkers used for the purpose of disease diagnosis, treatment, and follow-up. Furthermore, understanding interocular symmetry may play an important role in the diagnostic and therapeutic processes of a variety of other diseases in addition to SSc (23). It could also contribute to future study designs, as it remains unknown whether a randomly chosen eye of a patient is representative.

## Patients and methods

This prospective, single-center, cross-sectional study was conducted at the Ophthalmology Department of the Medical University of Bialystok between 2021 and 2024. The study protocol was approved by the local Bioethics Committee of the Medical University of Bialystok (decision no. APK.002.109.2021). This study was conducted in accordance with the principles of the Declaration of Helsinki. Written informed consent was obtained from each participant before enrollment in the study.

A total of 66 eyes from 33 patients with SSc admitted to the Department of Rheumatology and Internal Diseases of the Medical University of Bialystok were enrolled in the study. Diagnoses were made according to the 2013 ACR/EULAR SSc criteria (29) and subtypes were ascertained as diffuse or limited.

The control group comprised 80 eyes of 40 ophthalmologically and systemically healthy (self-reported) subjects undergoing routine ophthalmological assessments. Patients with SSc and controls did not differ in terms of age, sex, and axial length (AXL). All participants underwent ophthalmological examination, including refraction, best corrected visual acuity (BCVA) in Snellen converted to log MAR equivalents, intraocular pressure (IOP) measured using a Pascal dynamic contour tonometer (DCT; Zeimer Ophthalmic Systems AG, Port, Switzerland), slit-lamp biomicroscopy, AXL measured using a Tomey OA-2000 biometer (Nagoya, Japan), dilated fundus examination, and spectral-domain optical coherence tomography (SD-OCT, Heidelberg Engineering GmbH, Heidelberg, Germany; 2016). Blood pressure was measured immediately prior to obtaining OCT images after 5 min of rest in a sitting position.

Data regarding age, sex, disease duration, autoantibody profile, C-reactive protein (CRP) level, erythrocyte sedimentation rate (ESR; after 2 h), current smoking status, and details of systemic treatment were recorded. History of digital ulcers (present or past), cardiac involvement (elevated N-terminal pro b-type natriuretic peptide [NT-proBNP]) or heart fibrosis diagnosed using magnetic resonance imaging (MRI), diagnosis of interstitial lung disease (ILD) as confirmed by high-resolution computed tomography (HRCT) of the lungs, and joint involvement (arthralgia or joint swelling) were also included in the analysis. Nailfold capillaroscopy (NFC) was performed by means of a CapillaryScope 200 Dino-lite Digital microscope (MEDL4N PRO capillaroscopy equipment) and stratified as an ‘early’, ‘active’, or ‘late’ SSc pattern based on capillaroscopic characteristics (abnormal capillary morphology, capillary density, capillary dimension, and presence or absence of hemorrhages) (30).

Exclusion criteria included fundus pathology, ametropia ≥3 diopters, phacoemulsification less than 12 months prior to examination, history of posterior segment surgery, diabetes, retinal laser treatment, and poor quality of the OCT scans.

The sample size was calculated for the primary endpoint in the study—intraclass correlation (ICC). Assuming significance level of 5%, and power of 80%, the study would require 28 patients per group to confirm ICC between both eyes of 0.5 or higher as statistically significant. For both groups we have decided to collect data from a few additional patients, to make up for any potential loss of data or missing data (this way final sample for SSc group was 33 patients and 40 patients for controls).

### OCT image acquisition and analysis

The OCT images were taken in mydriasis within the same time interval (12 p.m.–3 p.m.) to avoid diurnal variations in choroidal thickness. The images were analyzed using Heidelberg Spectralis software (Heidelberg Engineering, Heidelberg, Germany) and ImageJ public domain software^1^ in conformity with the protocol previously described by Sonoda et al. and Agrawal et al. (31, 32) with some modifications. The most important adjustment was made to set the scale considering the stretching of the images to avoid erroneous quantification of the measured area (33). An image displayed with a 1:1 pixel aspect ratio (stretched axially) provides a better detailed visualization of a structure than a 1 × 1 μm image (OCT sampling density is higher in the axial direction than in the transverse direction) (33). Therefore, the scale was set by considering the pixel aspect ratio to reflect the actual size of the measured area.

**Figure 1:**
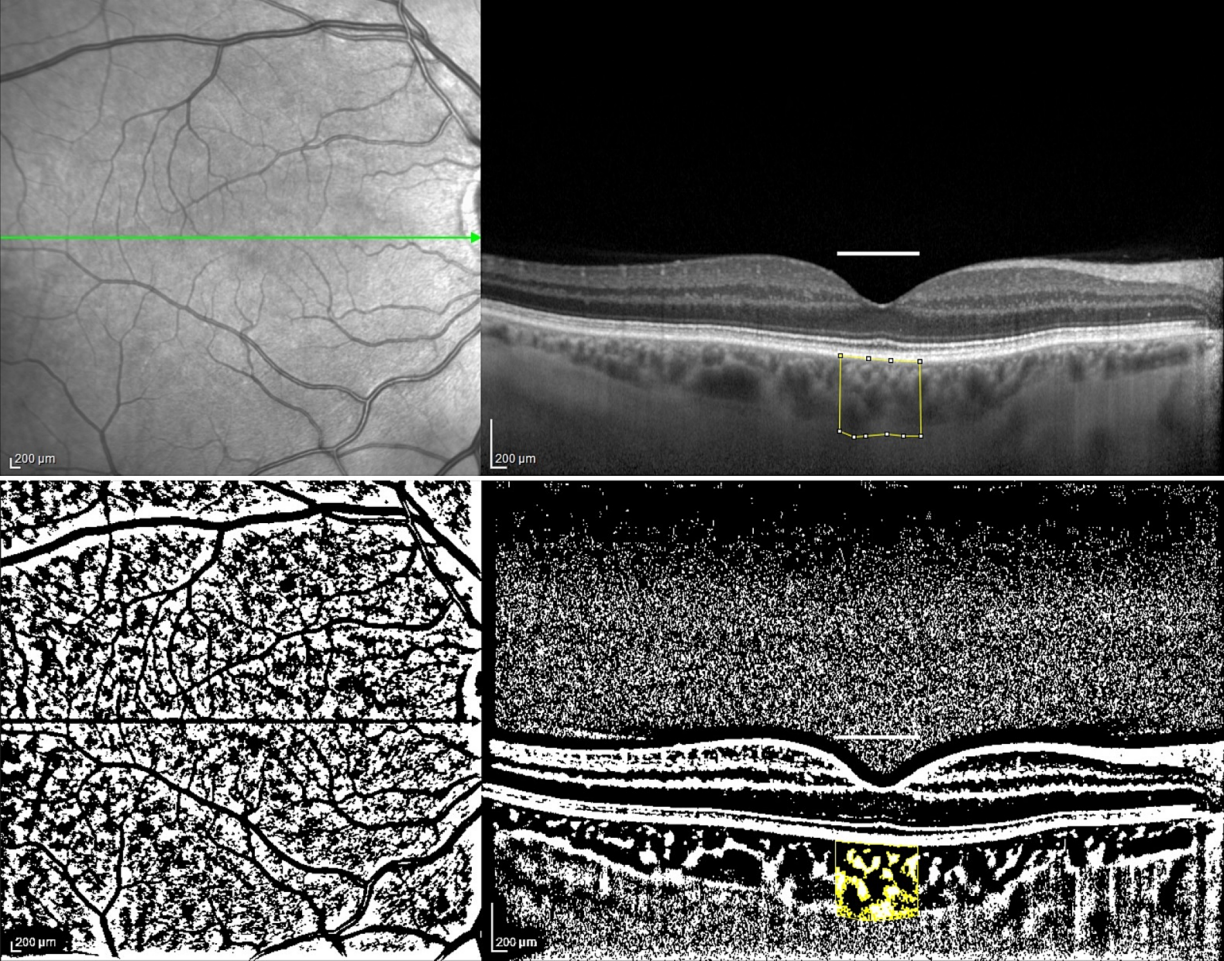
The enhanced depth imaging (EDI)-SD-OCT scans and choroidal binarization in a patient with systemic sclerosis (SSc). The upper panel shows an enhanced depth imaging (EDI)-SD-OCT scan of a patient with SSc, with marked examined total choroidal area (1,000 μm in width). The lower panel displays choroidal binarization, with the luminal area highlighted using a color threshold tool.

**Figure 2:**
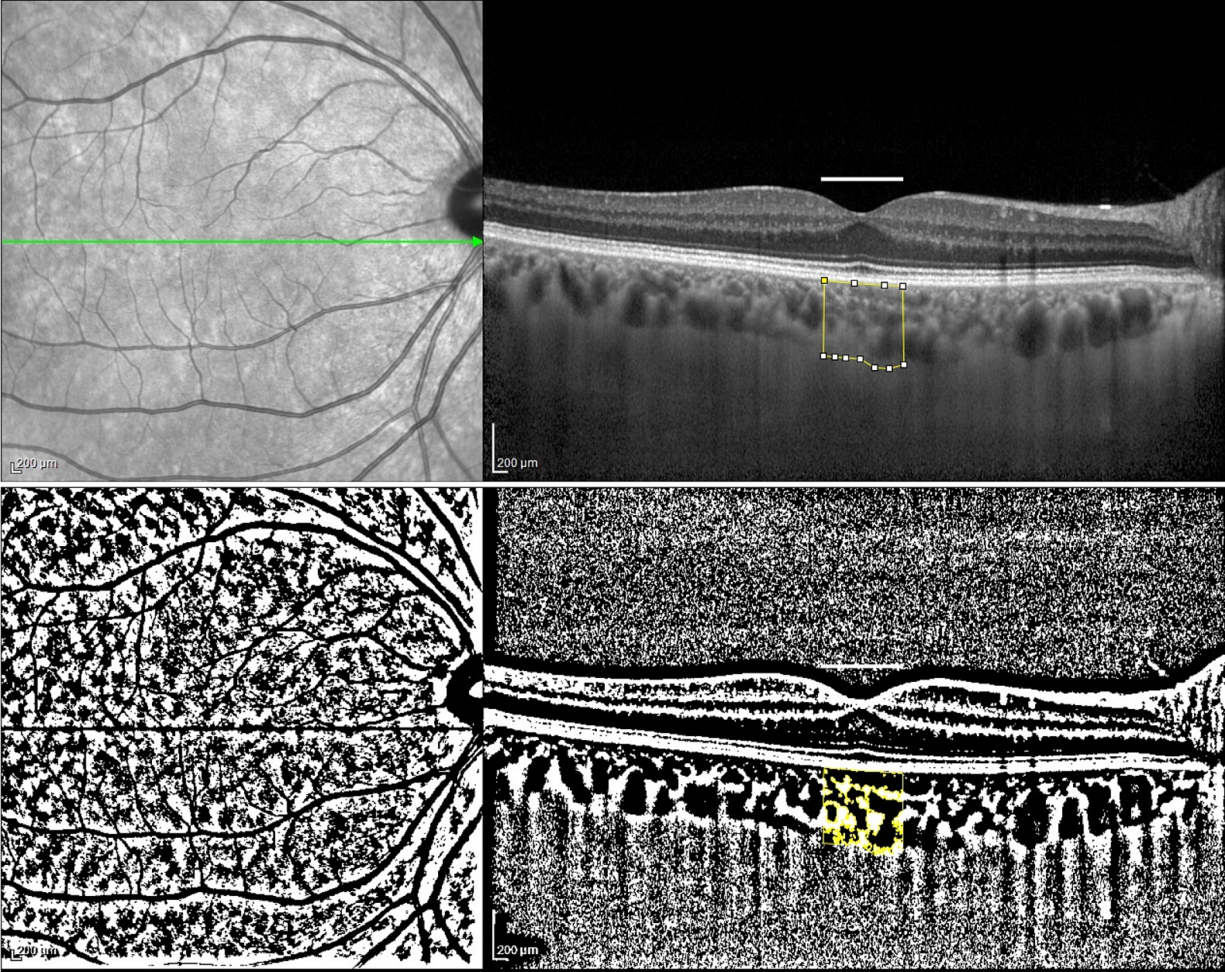
The enhanced depth imaging (EDI)-SD-OCT scans and choroidal binarization in a control patient. The upper panel shows an enhanced depth imaging (EDI)-SD-OCT scan of a patient belonging to the control group, with marked examined total choroidal area (1,000 μm in width). The lower panel displays choroidal binarization, with the luminal area highlighted using a color threshold tool.

The enhanced depth imaging (EDI)-SD-OCT imaging protocol for the macula comprised 25 raster scans (20° × 20°) and a linear 30° B-scan centered at the fovea. Choroidal thickness and volume were determined in the same manner as described in our previous studies (14, 15). Briefly, the internal limiting membrane (ILM) and Bruch’s membrane (BM) were detected automatically, while the choroidal–scleral junction (CSJ) was manually marked on each scan. Retinal parameters were calculated from the ILM to the BM and choroidal parameters from the BM to the CSJ. The average thickness and volume maps were created automatically according to the conventional ETDRS grid with nine subfields, including the central macular subfield (within a 500 μm radius) (34). Choroidal parameter values were calculated by subtracting the retinal parameters from the summed retinal and choroidal parameters. The SFCT was defined as the distance between the BM and CSJ at the fovea and was measured automatically. Binarization of the macular choroidal area was performed by two unblinded researchers (BP and AZ). The macular region was scanned using a single horizontal line scan (30°) centered on the fovea, with 100 frames averaged in a B-scan. As previously described (18) the measurement area was defined as 1,000 μm wide and centered on the fovea. The total choroidal area (TCA) was selected from the outer boundary of the RPE–BM layer to the CSJ using the polygon selection tool. The images were converted into 8-bit images to allow the application of the Niblack auto local threshold tool. The binarized images were reconverted into RGB images to allow the color threshold tool to be used for selecting dark pixels that represent vascularized areas. The luminal area (LA) and TCA were measured, while the stromal area (SA) was calculated by subtracting LA from TCA. The CVI was determined as the ratio of LA to TCA (%). The examples of one patient per group presenting EDI-OCT macular scans before and after binarization of the choroid are shown in Figures 1, 2.

**Figure 3:**
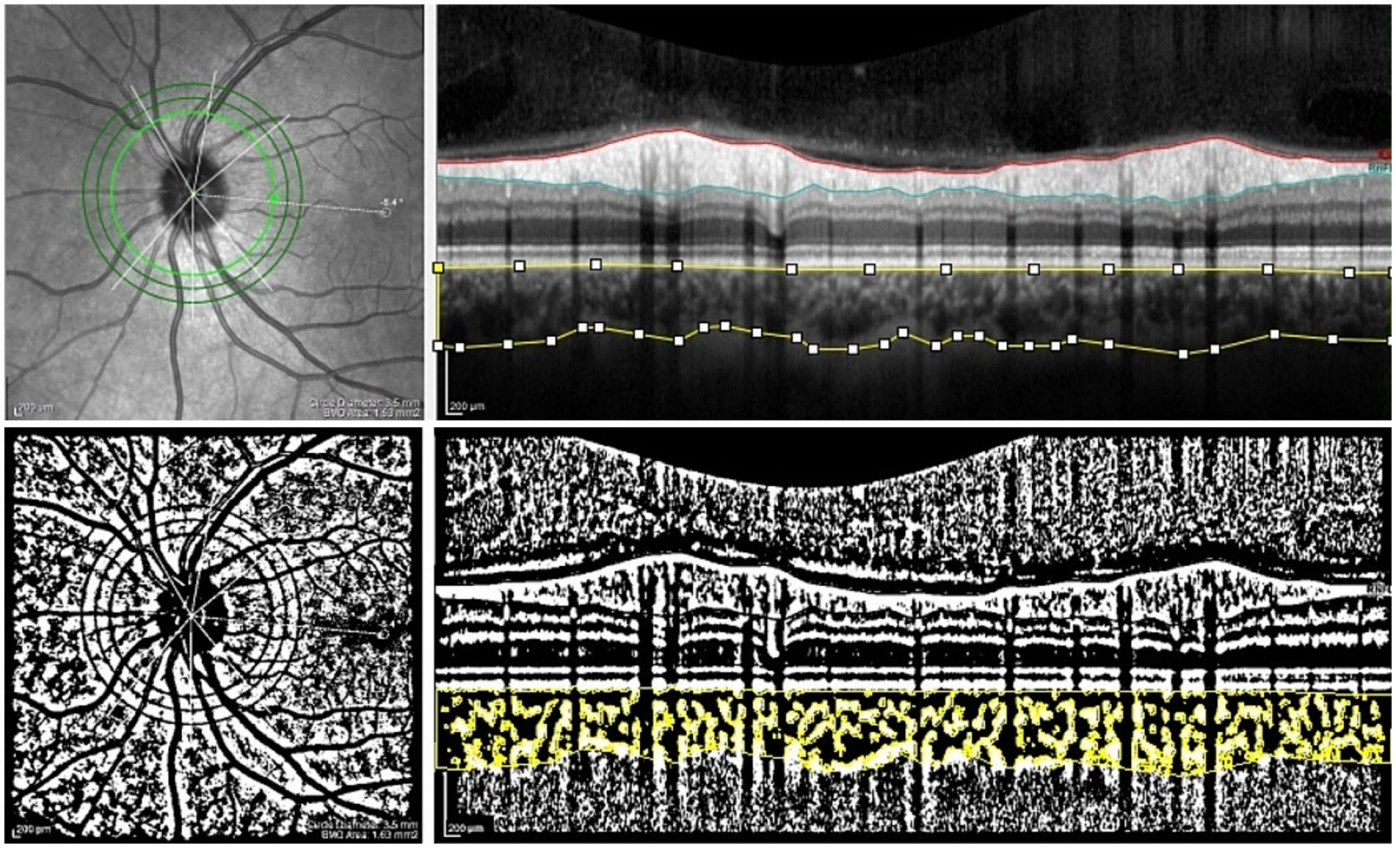
The peripapillary SD-OCT scans and choroidal binarization in a patient with systemic sclerosis (SSc). The upper panel shows an peripapillary SD-OCT scan of a patient with SSc. The lower panel displays choroidal binarization, with the luminal area highlighted using a color threshold tool.

**Figure 4:**
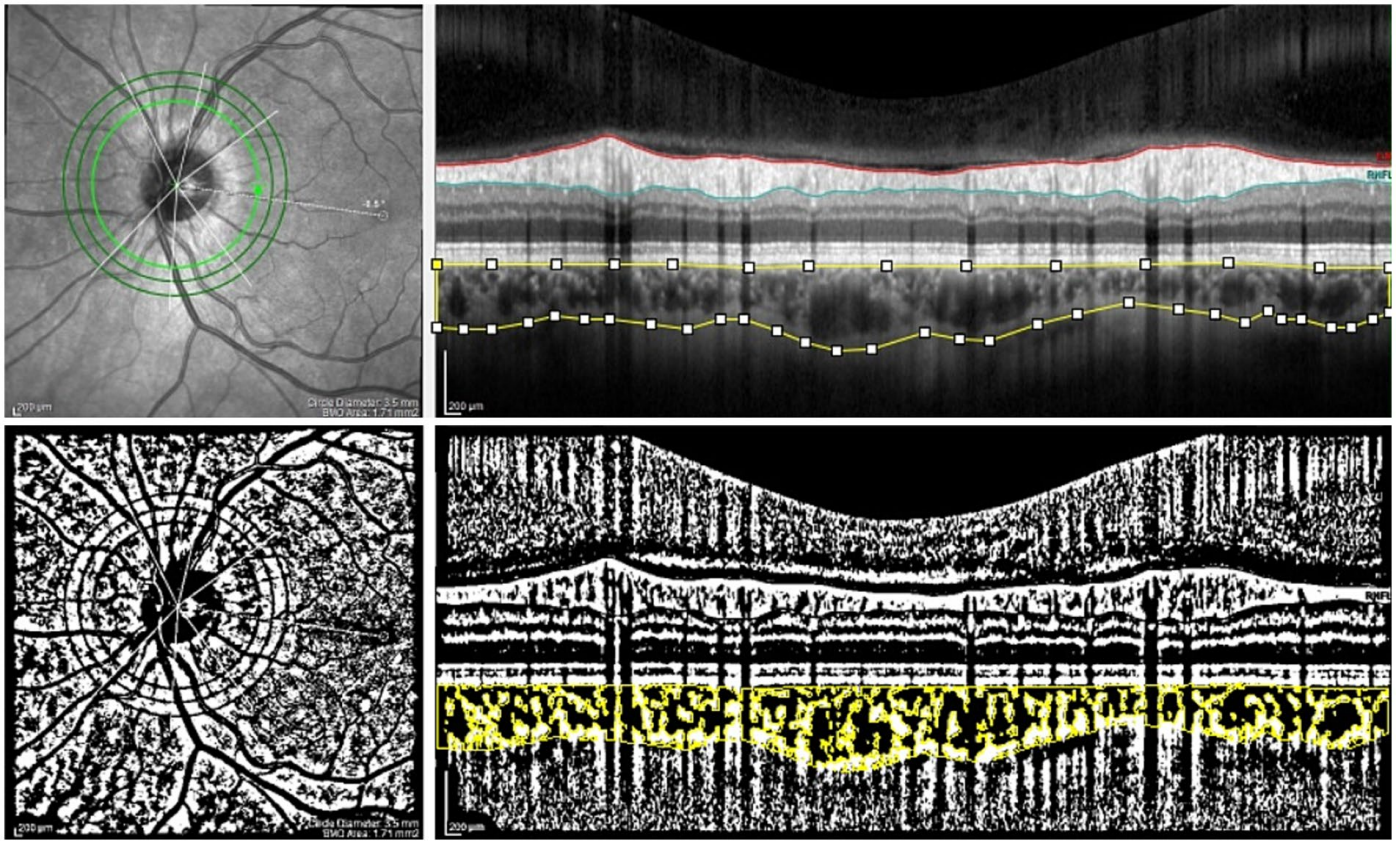
The peripapillary SD-OCT scans and choroidal binarization in a control patient. The upper panel shows an peripapillary SD-OCT scan of a patient belonging to the control group. The lower panel displays choroidal binarization, with the luminal area highlighted using a color threshold tool.

Peripapillary OCT images were obtained using a 3.5 mm diameter, 360°circle scan centered on the optic nerve head carried out with glaucoma software SD-OCT (Heidelberg Engineering, Heidelberg, Germany). This scan pattern was used to determine the following choroidal parameters: peripapillary choroidal thickness (pCT), peripapillary total choroidal area (pTCA), peripapillary luminal area (pLA), and the peripapillary stromal area (pSA). There is no automatic tool for pCT measurement; therefore, as previously described (19), the pCT was obtained by manually shifting the internal limiting membrane (ILM) to Bruch’s membrane (BM) and the retinal nerve fiber layer (RNFL) border to the CSJ. The results are presented as global and quadrant values (superior, inferior, temporal, and nasal) on a thickness map. Binarization of the peripapillary choroidal area was performed by two researchers (BP and PS). Briefly, pTCA was selected from the outer boundary of the RPE–Bruch’s membrane layer to the CSJ using the Polygon Selection tool. The following steps were similar to those used for the macular scan analysis. The peripapillary choroidal vascularity index (pCVI) was determined as the pLA to the pTCA ratio (%). The pSA was calculated by subtracting pLA from pTCA. The examples of one patient per group presenting peripapillary OCT images before and after binarization of the choroid are shown in Figures 3, 4.

The measurements were reviewed by the authors and disagreements were resolved through discussion. The inter-observer reproducibility of the measurements was assessed by measuring the intraclass correlation coefficient (ICC) and absolute agreement. The ICC values were high, and are presented in Table 1.

**Table 1:** The ICC values of choroidal parameters measurements between two raters.

	ICC	95% CI for ICC
mTCA	0.949	0.929–0.964
mLA	0.949	0.929–0.964
mSA	0.942	0.919–0.958
mCVI	0.875	0.828–0.910
pTCA	0.976	0.963–0.984
pLA	0.980	0.973–0.986
pSA	0.954	0.900–0.975
pCVI	0.853	0.723–0.913

CI, confidence interval; CVI, choroidal vascularity index; ICC, Intraclass correlation coefficient between 2 raters assuming two-way model and absolute agreement; LA, luminal area; m, macular; p, peripapillary; SA, stromal area; TCA, total choroidal area.

According to the related literature (35), the ICC values below 0.5 are indicative of poor reliability, the values between 0.5 and 0.75 indicate moderate reliability, the values between 0.75 and 0.9 indicate good reliability, and the values greater than 0.90 indicate excellent reliability.

### Statistical analysis

The analyses were performed using R 4.0.5. statistical software [R Core Team (2021). R: Language and Environment for Statistical Computing by the R Foundation for Statistical Computing, Vienna, Austria]. The normality of the distribution was validated using the Shapiro–Wilk test and based on skewness and kurtosis values. The data are presented as *n* (%) for nominal variables and as mean ± SD or median (Q1; Q3) for continuous variables. The groups were compared using the chi-square test or Fisher’s exact test for the nominal data and the t-test or Mann–Whitney U test for continuous variables, respectively. The interocular asymmetry in the choroidal parameters was quantified for SSc patients and controls using signed and absolute differences as per methodology by Lu et al. (27). The signed difference was calculated by subtracting the right eye value from the left eye value. The correlation analysis between the left and right eyes was based on the intraclass correlation coefficient (ICC), with a 95% confidence interval (CI) as a primary analysis (36). Additionally, as a secondary analysis, the relative mean difference between both eyes, Spearman’s correlation coefficient (*r*) between both eyes, and paired Wilcoxon test between both eyes (with Benjamini–Hochberg adjustments for multiple comparisons and without adjustment) were added. All the calculations were performed at *α* = 0.05.

The correlation between the two variables was quantified using a number (range: −1 and +1). According to Chan (37), the interpretation of the Spearman’s and Pearson’s correlation coefficient (*r*) is as follows: a value of 1 reflects perfect *r*, values 0.9–0.8 indicate very strong *r*, values 0.7–0.6 indicate moderate *r*, values 0.5–0.3 indicate fair *r* whereas values 0.2–0.1 indicate weak *r* and 0 indicates no correlation. Calculation of the sample size was made using ICC.Sample.Size package.

## Results

### Baseline characteristics

The demographic and clinical characteristics of the study groups are shown in Table 2.

**Table 2:** Demographic and clinical characteristics of the SSc patients and the control group.

Variable	Control group	SSc group	*p*
Number of patients	40	33	
Number of eyes, total	80	66	
Number of right/left eyes	40/40	33/33	
Age, years, mean ± SD	50.43 ± 10.52	50.97 ± 12.27	0.841
Sex, female, *n*, %	22 (55.0)	24 (72.7)	0.188
Sex, male, *n*, %	18 (45.0)	9 (27.3)	
MAP mmHg, mean ± SD	97.21 ± 12.43	86.47 ± 9.24	**<0.001**
Nicotine, *n*, %	6 (15.0)	3 (9.1)	0.484^a^
logMAR, median, (Q1; Q3)	0.00 (0.00; 0.00)	0.00 (0.00; 0.00)	0.686^b^
IOP, mmHg, mean ± SD	15.37 ± 2.13	13.94 ± 3.22	**0.007**
AXL, mm, mean ± SD	23.39 ± 0.97	23.15 ± 0.82	0.104
Duration of the disease, years, median, (Q1; Q3)	–	4.00 (2.00; 10.00)	–
Pulmonary involvement [Interstitial Lung Disease (ILD)/fibrosis], *n*, %	–	22 (66.7)	–
Cardiac involvement, *n*, %	–	11 (33.3)	–
Joint involvement, *n*, %	–	16 (48.5)	–
Digital ulcers present/history, *n*, %	–	11 (33.3)	–
CRP, mg/l, median (Q1; Q3)	–	1.45 (1.00; 3.43)	–
ESR, mm/2 h, median (Q1; Q3)	–	27.00 (18.00; 39.00)	–
SSc subtype limited/ diffuse n, number of patients	–	11/22	–
Anti-Scl70 positive, *n*, %	–	16 (53.3)	–
Anti- centromere positive, *n*, %	–	7 (23.3)	–
Other Abs positive, *n*, %	–	13 (43.3)	–
NFC, active/early/late; number of patients	–	15/9/9	–

Abs, antibodies; AXL, axial length; CRP, C-reactive protein; ESR, erythrocyte sedimentation rate; h, hours; IOP, intraocular pressure; MAP, mean arterial pressure; NFC, nailfold capillaroscopy; SSc, systemic sclerosis.

*p < 0.05 in bold font.*

Comparison between both groups was made with chi-square test or Fisher exact test^a^ for nominal data and with t-test or Mann–Whitney U test^b^ for continuous data.

No significant differences in age, sex, or nicotine intake were found between the SSc and control groups.

The patients differed in terms of mean arterial pressure (MAP), which was lower in patients with SSc than in the control group (*p* < 0.01). The studied groups differed in terms of mean IOP values; in patients with systemic sclerosis, the IOP was lower than that in controls (*p* = 0.007).

In both groups, the right and left eyes did not differ in terms of: 1. IOP: 13.95 ± 3.49 mmHg vs. 13.94 ± 3.01 mmHg, *p* > 0.999; 15.46 ± 2.41 mmHg vs. 15.28 ± 1.84 mmHg, *p* = 0.903, right vs. left eyes, the SSc group vs. controls, respectively; 2. AXL: 23.17 ± 0.85 mm vs. 23.13 ± 0.81 mm, *p* = 0.518; 23.37 ± 1.00 mm vs. 23.42 ± 0.95 mm, *p* = 0.908, right vs. left eyes, the SSc group vs. controls, respectively; 3. logMAR 0.01 ± 0.03 vs. 0.02 ± 0.06, *p* > 0.999; 0.02 ± 0.06 vs. 0.00 ± 0.02, *p* = 0.590, right vs. left eyes, the SSc group vs. controls, respectively.

### The interocular comparison of choroidal parameters in the SSc group and controls

Table 3 shows the values of the choroidal parameters of both eyes in the macular and peripapillary areas: choroidal thickness, choroidal volume, total choroidal area (TCA), luminal area (LA), stromal area (SA), and the choroidal vascularity index (CVI).

**Table 3:** Values of the choroidal parameters in the patients with the systemic sclerosis and controls.

Group	Variable	Mean ± SD left eyes	Mean ± SD right eyes	Median (Q1; Q3) left eyes	Median (Q1; Q3) right eyes	Relative mean difference (%) between left and right eyes
Systemic sclerosis patients	mTCA, μm^2^	316,828.71 ± 63,214.82	329,109.85 ± 65,529.12	319,288.75 (272,132.00; 350,998.00)	319,346.75 (295,882.88; 378,651.25)	−3.80
	mLA, μm^2^	213,623.14 ± 44,627.45	220,629.80 ± 41,210.45	216,480.25 (184,573.88; 237,467.63)	215,477.00 (197,717.63; 251,082.88)	−3.23
	mSA, μm^2^	103,205.57 ± 20,183.98	108,480.05 ± 26,441.43	103,420.00 (90,308.38; 114,712.38)	103,802.50 (89,663.38; 126,480.13)	−4.98
	mCVI, %	67.30 ± 2.44	67.23 ± 2.84	67.20 (66.07; 68.81)	67.15 (65.39; 69.23)	0.11
	Central macular choroidal thickness, μm	281.48 ± 61.63	291.60 ± 68.47	284.00 (237.00; 307.00)	284.00 (262.00; 344.00)	−3.53
	SFCT, μm	280.07 ± 63.45	297.47 ± 66.84	281.00 (236.00; 317.00)	295.00 (249.00; 349.50)	−6.02
	Central macular choroidal volume, μm^3^	0.22 ± 0.05	0.23 ± 0.05	0.22 (0.19; 0.24)	0.22 (0.21; 0.27)	−3.53
	Total choroidal volume, μm^3^	7.33 ± 1.31	7.36 ± 1.65	7.37 (6.45; 7.93)	7.38 (6.29; 8.32)	−0.36
	pTCA, μm^2^	2,439,386.76 ± 684,051.36	2,363,522.97 ± 705,717.37	2,429,580.50 (2,013,428.50; 3,069,290.00)	2,320,543.25 (1,838,453.50; 2,861,731.75)	3.16
	pLA, μm^2^	1,577,668.52 ± 457,033.97	1,518,730.13 ± 478,359.82	1,547,415.50 (1,248,618.00; 1,969,349.00)	1,465,544.50 (1,164,621.13; 1,869,769.63)	3.81
	pSA, μm^2^	861,718.24 ± 232,204.62	844,792.83 ± 233,833.13	882,731.50 (702,763.50; 1,046,614.00)	806,799.50 (688,120.38; 972,602.63)	1.98
	pCVI, %	64.54 ± 1.68	63.93 ± 2.18	64.48 (63.61; 65.75)	64.36 (62.31; 65.22)	0.94
	pCT Global, μm	193.58 ± 62.24	189.24 ± 57.50	185.00 (154.00; 242.00)	182.00 (143.00; 235.00)	2.26
	pCT S, μm	199.73 ± 60.15	202.79 ± 62.90	196.00 (162.00; 250.00)	193.00 (155.00; 255.00)	−1.52
	pCT I, μm	177.76 ± 67.44	170.24 ± 54.88	181.00 (117.00; 226.00)	160.00 (132.00; 209.00)	4.32
	pCT T, μm	203.58 ± 75.02	195.64 ± 67.04	196.00 (148.00; 263.00)	184.00 (154.00; 249.00)	3.98
	pCT N, μm	193.30 ± 59.03	188.52 ± 54.80	200.00 (146.00; 232.00)	181.00 (151.00; 227.00)	2.51
Control group	mTCA, μm^2^	359,498.50 ± 80,749.59	361,454.75 ± 97,205.21	359,688.25 (304,275.38; 415,141.25)	366,920.75 (294,563.13; 416,039.75)	−0.54
	mLA, μm^2^	238,152.57 ± 49,081.47	238,491.59 ± 63,553.61	238,283.75 (201,310.00; 267,542.25)	249,230.50 (195,780.75; 272,242.38)	−0.14
	mSA, μm^2^	121,345.93 ± 33,467.39	122,963.16 ± 35,675.06	119,868.50 (98,719.63; 143,941.50)	118,384.25 (98,138.00; 152,267.75)	−1.32
	mCVI, %	66.57 ± 3.01	66.02 ± 2.62	65.97 (64.31; 68.98)	65.67 (64.29; 67.25)	0.83
	Central macular choroidal thickness, μm	322.33 ± 72.67	314.48 ± 91.30	326.00 (275.50; 368.50)	311.00 (254.50; 383.25)	2.47
	SFCT, μm	321.51 ± 74.34	315.63 ± 101.82	304.00 (272.50; 374.50)	294.00 (242.25; 390.50)	1.85
	Central macular choroidal volume, μm^3^	0.25 ± 0.06	0.25 ± 0.07	0.25 (0.22; 0.29)	0.24 (0.20; 0.30)	2.03
	Total choroidal volume, μm^3^	7.86 ± 1.68	7.98 ± 1.86	7.95 (6.66; 8.87)	7.88 (6.59; 8.93)	−1.46
	pTCA, μm^2^	2,504,610.85 ± 598,789.90	2,592,788.89 ± 668,209.81	2,399,093.25 (2,160,643.38; 2,815,545.50)	2,579,502.25 (2,009,824.00; 3,068,943.38)	−3.46
	pLA, μm^2^	1,646,654.48 ± 411,147.00	1,710,499.89 ± 452,159.66	1,595,197.25 (1,402,041.75; 1,879,013.50)	1,716,502.50 (1,331,180.38; 2,033,625.88)	−3.80
	pSA, μm^2^	857,956.38 ± 196,663.12	882,289.00 ± 225,231.30	853,966.25 (715,619.63; 952,597.38)	822,028.50 (705,969.50; 1,055,796.38)	−2.80
	pCVI, %	65.62 ± 2.12	65.83 ± 2.13	65.74 (64.28; 66.75)	65.54 (64.50; 67.16)	−0.32
	pCT Global, μm	199.38 ± 49.52	206.73 ± 58.72	192.00 (164.75; 236.00)	202.50 (156.50; 242.25)	−3.62
	pCT S, μm	211.33 ± 54.60	225.15 ± 62.10	203.50 (167.00; 248.25)	216.00 (176.75; 263.50)	−6.33
	pCT I, μm	175.88 ± 49.59	178.98 ± 60.58	168.50 (143.25; 198.25)	171.00 (129.25; 211.25)	−1.75
	pCT T, μm	210.08 ± 51.62	216.65 ± 61.45	205.50 (174.25; 253.25)	215.00 (175.00; 252.75)	−3.08
	pCT N, μm	200.35 ± 53.00	206.53 ± 65.36	190.00 (164.50; 229.50)	197.00 (158.50; 257.00)	−3.04

CT, choroidal thickness; CVI, choroidal vascularity index; I, inferior; LA, luminal area; m, macular; N, nasal; p, peripapillary; Q1, quartile 1; Q3, quartile 3; S, superior; SA, stromal area; SD, standard deviation; SFCT, subfoveal choroidal thickness; T, temporal; TCA, total choroidal area.

Relative mean difference calculated as per formula: (left eye − right eye)/average from both eyes * 100.

The overall choroidal thickness parameters in both groups showed interocular symmetry (Table 4). Signed and absolute interocular differences in the choroidal parameters for SSc patients and controls are presented in in the Supplementary Tables 1, 2. The normal 95% limits of differences can be used as a reference point for physiological asymmetry, i.e., values below 2.5% point/above 97.5% point for signed differences or values above 95% limits for signed differences can be treated as abnormal.

**Table 4:** Comparison of the choroidal parameters in fellow eyes.

Group	Variable	ICC between both eyes	95% CI for ICC	*r* between both eyes	*p* value for *r*	*p*-value of Wilcoxon test (w/o correction)^a^	*p*-value of Wilcoxon test (B-H correction)^a^
Systemic sclerosis patients	mTCA	0.725	0.490–0.862	0.650	**<0.001**	0.913	>0.999
	mLA	0.730	0.496–0.865	0.650	**<0.001**	0.913	>0.999
	mSA	0.674	0.412–0.834	0.660	**<0.001**	0.913	>0.999
	mCVI	0.555	0.230–0.767	0.588	**0.001**	>0.999	>0.999
	Central macular choroidal thickness	0.723	0.484–0.863	0.686	**<0.001**	0.913	>0.999
	SFCT	0.720	0.476–0.862	0.726	**<0.001**	0.913	>0.999
	Central macular choroidal volume	0.704	0.453–0.852	0.686	**<0.001**	0.913	>0.999
	Total choroidal volume	0.882	0.758–0.945	0.867	**<0.001**	0.913	>0.999
	pTCA	0.924	0.849–0.963	0.926	**<0.001**	0.191	0.384
	pLA	0.926	0.850–0.964	0.923	**<0.001**	0.135	0.384
	pSA	0.901	0.803–0.951	0.899	**<0.001**	0.299	0.384
	pCVI	0.629	0.349–0.805	0.677	**<0.001**	*0.015*	0.135
	pCT Global	0.931	0.866–0.965	0.934	**<0.001**	0.256	0.384
	pCT S	0.906	0.820–0.953	0.887	**<0.001**	0.344	0.387
	pCT I	0.873	0.759–0.935	0.905	**<0.001**	0.217	0.384
	pCT T	0.925	0.852–0.962	0.920	**<0.001**	0.085	0.383
	pCT N	0.825	0.676–0.909	0.852	**<0.001**	0.514	0.514
Control group	mTCA	0.776	0.609–0.877	0.772	**<0.001**	0.957	0.976
	mLA	0.757	0.579–0.866	0.694	**<0.001**	0.976	0.976
	mSA	0.781	0.618–0.880	0.805	**<0.001**	0.957	0.976
	mCVI	0.555	0.294–0.740	0.536	**0.001**	0.957	0.976
	Central macular choroidal thickness	0.759	0.586–0.866	0.760	**<0.001**	0.957	0.976
	SFCT	0.645	0.415–0.797	0.697	**<0.001**	0.957	0.976
	Central macular choroidal volume	0.754	0.578–0.863	0.762	**<0.001**	0.957	0.976
	Total choroidal volume	0.865	0.758–0.926	0.826	**<0.001**	0.957	0.976
	pTCA	0.891	0.800–0.942	0.909	**<0.001**	0.150	0.425
	pLA	0.891	0.797–0.942	0.911	**<0.001**	0.087	0.392
	pSA	0.851	0.737–0.918	0.888	**<0.001**	0.354	0.561
	pCVI	0.508	0.236–0.706	0.441	**0.005**	0.420	0.561
	pCT Global	0.872	0.769–0.930	0.883	**<0.001**	0.189	0.425
	pCT S	0.788	0.619–0.885	0.824	**<0.001**	*0.023*	0.207
	pCT I	0.839	0.716–0.911	0.812	**<0.001**	0.615	0.615
	pCT T	0.816	0.680–0.898	0.808	**<0.001**	0.595	0.615
	pCT N	0.816	0.680–0.898	0.820	**<0.001**	0.436	0.561

CI, confidence interval; CT, choroidal thickness; CVI, choroidal vascularity index; I, inferior; ICC, intraclass correlation coefficient; LA, luminal area; m, macular; N, nasal; p, peripapillary; r, Spearman correlation coefficient; S, superior; SA, stromal area; SFCT, subfoveal choroidal thickness; T, temporal; TCA, total choroidal area.

*p < 0.05 in bold font.*

Wilcoxon test comparing average level between left eyes and right eyes.

a *p*-value after Benjamini-Hochberg correction for multiple comparisons. The correction was made separately for macular (8 comparisons) and peripapillary (9 comparisons) choroidal parameters.

In the SSc and control groups, the intraclass correlation coefficient (ICC) (i.e., how data from one eye overlapped with data from the other eye) for selected choroidal parameters ranged from moderate to good in the macular region and from moderate to excellent in the peripapillary region. The degree of correlation was higher in patients with SSc than in the control group.

The parameter that showed the lowest correlation among those examined was CVI—in both groups, as well as in both examined areas.

Spearman’s correlation coefficient (*r*), which represents a linear relationship between the right and left eyes, showed a strong correlation (moderate to very strong) for most parameters, except for the CVI in the submacular area (mCVI) in both groups, where the correlation was satisfactory (fair): in the SSC group (*r* = 0.588, *p* = 0.001) and in the control group (*r* = 0.536, *p* = 0.001). The peripapillary CVI (pCVI) in the SSc group showed moderate interocular correlation (*r* = 0.677, *p* < 0.001), but in the control group showed only satisfactory interocular correlation (*r* = 0.441, *p* = 0.005). In general, the interocular correlation of choroidal parameters was stronger in the peripapillary area than in the macular area in both groups.

The analysis conducted with the Wilcoxon test to determine whether the mean level for the left eye differs significantly from that for the right eye, following the Benjamini-Hochberg correction for multiple comparisons, was not statistically significant for any of the assessed parameters.

Further analyses of the interocular symmetry of the choroidal parameters in both groups were conducted based on sex. Moreover, the comparisons were carried out in the subgroups of the SSc patients stratified according to SSc subtype, Scl70 antibody positivity and previous and/or active digital ulcers (Supplementary Tables 3.1–6.2). Single parameters lost their statistical significance after taking into account the analyses of less numerous groups. However, this did not affect the overall results and their interpretation.

## Discussion

In this study, we comprehensively compared choroidal parameters between fellow eyes in the macular and peripapillary regions in patients with SSc and controls. We demonstrated that the choroidal parameters (choroidal thickness, choroidal volume, and CVI) were symmetrical between the eyes in both study groups.

Despite anatomical differences in the vascularization of the right and left eyes (as the left common carotid artery comes from the aortic arch, and the right one from the brachiocephalic trunk) and despite single reports on interocular choroidal asymmetry (27), the symmetry of choroidal parameters in healthy patients has been widely described in the related literature (23–28). We have even been able to demonstrate a high degree of interocular symmetry at the metabolomic level (38).

This finding is consistent with our results. We have shown the interocular correlation of choroidal parameters in SSc patients and controls. In our analysis, we did not find any significant interocular differences in terms of CT in either study group, whereas the CT in the SSc group was found to be smaller than that in the control group (18). This result confirms the impact of SSc on choroidal parameters, symmetrically in both eyes of the patient.

The CVI between the eyes is symmetrical, although it shows the lowest interocular correlation between eyes among the parameters we tested, which is surprising because the CVI is considered a rather reliable and stable parameter (as opposed to CT) owing to its low susceptibility to the influence of other factors (12). This result in controls is consistent with the results of Lu et al. (27), who found that unlike a strong interocular correlation in the macular choroidal thickness, there was only a moderate interocular correlation in CVI in healthy subjects.

In our subgroup analyses, in general, we did not find significant differences of the choroidal parameters depending on sex in both groups, neither depending on SSc subtype, Scl70 antibody positivity and previous and/or active digital ulcers in SSc patients. These results may confirm consistency within different datasets. Albeit, one needs to keep in mind the relatively small sample size of the subgroups, consistent with the numbers in previous studies though (8, 9, 39, 40).

When designing the study, we hypothesized that there might be interocular asymmetry in choroidal involvement due to the multifactorial and complex impact of SSc on the vessels. Microcirculatory disturbances in the retina and choroid are triggered by a specific immune response and may correlate with the activity and severity of the disease (41). Histopathological examination of blood vessels within the choroid revealed a number of abnormalities, such as damage to endothelial cells, thickening of the basement membrane, lack of pericytes, and accumulation of abnormal material inside and around endothelial cells (6, 42) resulting in areas of non-perfusion in the choroidal capillary bed (43). Damage to the retinal pigment epithelium, probably due to the involvement of choroidal blood vessels, has been previously described (44, 45). Ingegnoli et al. (46) also drew attention to the impaired control of the tone of blood vessels in the choroid in patients with SSc, which may result in damage to the optic nerve and cause normal-tension glaucoma. A study assessing the parameters of the right common carotid artery in patients with SSc showed greater arterial stiffness than in the control group (47). A decreased choroidal thickness (CT) in the patients with the systemic sclerosis was also demonstrated by Esen et al., and Coşkun et al. (48, 49). Coşkun et al. claimed that vasculopathy in patients with SSc may lead to choroidal atrophy. Microcirculation in the eye is already affected in the early phase of the disease, and patients displaying Raynaud’s phenomenon, which may be the first symptom of SSc, show a decrease in the thickness of the choroid (46). SSc is the most common connective tissue disease causing Raynaud’s syndrome, that is, a transient, vasospastic phenomenon, affecting the fingers and toes. Secondary Raynaud syndrome is characterized by ischemic skin lesions, capillaroscopic abnormalities, and asymmetric attacks, which distinguish it from the other symmetrical symptoms mentioned above (49). All of the above-mentioned data might support our provisional hypothesis of interocular asymmetry. However, this finding was not confirmed in the present study.

In terms of symmetry of involvement, when other organs are considered, SSc may also be associated with idiopathic inflammatory myopathies, characterized by symmetrical weakness of proximal muscles (50). In patients with SSc with musculoskeletal symptoms, whole-body MRI usually shows symmetric involvement of muscles, joints, fascia, and tendon entheses, which corresponds to our results (51). Furthermore, digital ulcers are observed in more than half of the patients with SSc (52). In the course of some connective tissue diseases, a condition called mechanic’s hand often occurs. Eighty % of the examined patients with mechanic’s hands displayed the SSc pattern on the NFC test. During this disease, hyperkeratotic eruptions appear along the fingers. They are bilateral and symmetrical (53).

As far as the methodology of this study is concerned, in our previous studies (18, 19) we found no significant differences in macular and peripapillary choroidal parameters between the SSc subtypes: diffuse cutaneous systemic sclerosis (dcSSc) and limited cutaneous systemic sclerosis (lcSSc) (*p* > 0.05). Therefore, in this study, all patients with SSc were treated as a single group. The study groups were matched for age, sex, and nicotine consumption. They differ only in terms of MAP, which is lower in patients with the systemic sclerosis than in controls. The studied groups differed in terms of IOP in patients with systemic sclerosis, the IOP is lower than in the healthy controls. One of the reasons might be altered corneal biomechanical properties in patients with SSc as described by Emre et al. (54), despite the fact that the central corneal thickness did not differ between the groups (unpublished data). We measured IOP using a Pascal dynamic contour tonometer (DCT; Zeimer Ophthalmic Systems AG, Port, Switzerland). Unfortunately, there is no data available in literature whether this method of assessment might be not appropriate in patients with SSc. Furthermore, SSc patients were taking antihypertensive medications (often calcium channel blockers) and phosphodiesterase inhibitors (for digital ulcers), which lower blood pressure, and yet it turns out that systolic and diastolic blood pressure affect IOP (55–57). Consequently, since they had lower MAP (mean arterial pressure), one may assume that they probably had consistently lower IOP as well. Even though IOP and MAP may be related to choroidal parameters, in this study, there was no statistical difference in IOP between the right and left eyes. Furthermore, we assumed that the effect of MAP as a systemic factor was similar in both eyes. The data from the related literature regarding differences in the choroidal parameters between patients with SSc and controls (17–19, 39, 40, 46, 48, 49) do not apply to this study, as its aim has been the inter-eye study within the groups (separately for SSc and controls) instead of the intergroup comparison (so not SSc versus controls).

A limitation of our study is the relatively small number of patients, which is due to the rarity of systemic sclerosis and the sample size was partly determined by the number of participants who met our inclusion criteria. However, this number is in accordance with other relevant studies (8, 9, 39, 40). Our results would need to be validated in a larger population to allow for multiple comparisons and subgroup analyses. With regard to the analysis of OCT scans, the shadowing effect may play a role in the CVI assessment, particularly in peripapillary CVI evaluation. However, neither have the inventors of the CVI (Agrawal et al. and Sonoda et al.) (12, 31) compensated for this nor is currently any software available to overcome that problem.

This study has several strengths. To the best of our knowledge, this is the first study to compare selected choroidal parameters between the eyes of patients with SSc. This is a prospective study. In addition, we comprehensively examine the parameters of the choroid in the submacular and peripapillary areas—the analysis of the features in the peripapillary area is an innovative issue in the case of SSc. This is of particular interest, as patients with SSc have an increased risk of developing glaucoma (8–10). Moreover, in previous studies, the assessment of choroidal thickness was based on measurements at single and different points, whereas in this study, we assessed the macular and peripapillary areas more comprehensively. Additionally, we have included in the research a quite rare parameter, that is, the volume of the choroid, and a relatively new index, the CVI, a stable parameter characterizing the choroid in detail. OCT was performed at a similar time of the day, which excluded its influence on the results.

In terms of future directions, longitudinal studies to evaluate the changes in choroidal parameters as the disease progresses may be valuable both in the macular and peripapillary regions. It would be interesting to observe the changes in the context of their interocular symmetry, not only juxtaposed with the clinical parameters of SSc, but also with changes in the RNFL, knowing the increased risk of glaucoma development in these patients. Our results would need to be validated in a different cohort of SSc patients as asymmetry might occur if these patients start exhibiting the ocular pathology such as glaucoma.

The concurrent analysis of SD-OCT scans and OCT angiography scans would provide a wider perspective in two dimensions. Indocyanine green angiography would be a valuable complementary tool to characterize the choroidal vasculature. Adding ultrasound of the retrobulbar vessels and internal carotid artery would broaden our knowledge of blood supply to the eyeball in patients with SSc.

## Conclusion

Our study provides insights into the pathophysiology of the choroid in patients with SSc. Choroidal characteristics such as macular and peripapillary CT, choroidal volume, and CVI are symmetrical for fellow eyes in patients with SSc as well as in controls. This suggests that the SSc affects both eyes concurrently and symmetrically. These parameters from one eye are representative of those of the fellow eye in the given patient. However, the mCVI and pCVI are more variable than the CT and volume. Our conclusions are relevant to the described and probably similar cohorts but would need to be validated in different SSc cohorts depending on the characteristics of SSc as well as ocular manifestations. This conclusion may contribute to the design of future studies and their proper interpretation as well as broaden our knowledge of the pathophysiology of SSc.

## Data Availability

The raw data supporting the conclusions of this article will be made available by the authors without undue reservation.

## Footnotes

^1^ https://imagej.nih.gov/ij/

## References

[1] Sakkas LI. New developments in the pathogenesis of systemic sclerosis. Autoimmunity. (2005) 38:113–6. doi: 10.1080/16066350500095415, PMID: 16040330

[2] Rabquer BJ Koch AE. Angiogenesis and vasculopathy in systemic sclerosis: evolving concepts. Curr Rheumatol Rep. (2012) 14:56–63. doi: 10.1007/s11926-011-0219-1, PMID: 22083296

[3] Tailor R Gupta A Herrick A Kwartz J. Ocular manifestations of scleroderma. Surv Ophthalmol. (2009) 54:292–304. doi: 10.1016/j.survophthal.2008.12.007, PMID: 19298906

[4] Gomes BAF Santhiago MR Magalhães P Kara-Junior N de Azevedo MNL Moraes HV Jr. Priscilla Magalhães, Newton Kara-Junior, Mário N L de Azevedo, Haroldo V Moraes Jr

[5] Hesse RJ Slagle DF. Scleroderma choroidopathy: report of an unusual case. Ann Ophthalmol. (1982) 14:524–5. PMID: 7114687

[6] Farkas TG Sylvester V Archer D. The choroidopathy of progressive systemic sclerosis (scleroderma). Am J Ophthalmol. (1972) 74:875–86. doi: 10.1016/0002-9394(72)91208-1, PMID: 4264778

[7] Pieklarz B Gińdzieńska-Sieśkiewicz E Zawadzka I Bagrowska M Daniluk J Konopińska J. Purtscher-like retinopathy in a patient with systemic sclerosis: a case report and narrative review. Biomedicines. (2023) 11:839. doi: 10.3390/biomedicines11030839, PMID: 36979818 PMC10044861

[8] Agapito Tito CV Silvatti J de Almeida INF Taniguchi EV Prata TS Paranhos A Jr. Structural abnormalities associated with glaucoma using swept-source optical coherence tomography in patients with systemic sclerosis. Int Ophthalmol. (2022) 42:1369–80. doi: 10.1007/s10792-021-02124-1, PMID: 34822051

[9] Sahin-Atik S Koc F Akin-Sari S Ozmen M. Retinal nerve fiber and optic disc morphology using spectral-domain optical coherence tomography in scleroderma patients. Eur J Ophthalmol. (2017) 27:281–4. doi: 10.5301/ejo.5000827, PMID: 27445077

[10] Allanore Y Parc C Monnet D Brézin AP Kahan A. Increased prevalence of ocular glaucomatous abnormalities in systemic sclerosis. Ann Rheum Dis. (2004) 63:1276–8. doi: 10.1136/ard.2003.013540, PMID: 15361386 PMC1754752

[11] Pichi F Aggarwal K Neri P Salvetti P Lembo A Nucci P. Choroidal biomarkers. Indian J Ophthalmol. (2018) 66:1716–26. doi: 10.4103/ijo.IJO_893_18, PMID: 30451172 PMC6256910

[12] Agrawal R Gupta P Tan KA Cheung CMG Wong TY Cheng CY. Choroidal vascularity index as a measure of vascular status of the choroid: measurements in healthy eyes from a population-based study. Sci Rep. (2016) 6:21090. doi: 10.1038/srep21090, PMID: 26868048 PMC4751574

[13] Raciborska A Sidorczuk P Konopińska J Dmuchowska DA. Interocular symmetry of choroidal parameters in patients with diabetic retinopathy with and without diabetic macular edema. J Clin Med. (2023) 13:176. doi: 10.3390/jcm13010176, PMID: 38202183 PMC10779809

[14] Sidorczuk P Pieklarz B Konopinska J Saeed E Mariak Z Dmuchowska D. Foveal avascular zone does not correspond to choroidal characteristics in patients with diabetic retinopathy: a single-center cross-sectional analysis. Diabetes Metab Syndr Obes. (2021) 14:2893–903. doi: 10.2147/DMSO.S318860, PMID: 34234487 PMC8254029

[15] Dmuchowska DA Sidorczuk P Pieklarz B Konopińska J Mariak Z Obuchowska I. Quantitative assessment of choroidal parameters in patients with various types of diabetic macular oedema: a single-centre cross-sectional analysis. Biology (Basel). (2021) 10:725. doi: 10.3390/biology10080725, PMID: 34439957 PMC8389323

[16] Steiner M Esteban-Ortega MDM Muñoz-Fernández S. Choroidal and retinal thickness in systemic autoimmune and inflammatory diseases: a review. Surv Ophthalmol. (2019) 64:757–69. doi: 10.1016/j.survophthal.2019.04.007, PMID: 31034855

[17] Ataş F Kaya M Ayhan Z Ozkan O Birlik M. Evaluation of choroidal vascularity index in systemic sclerosis patients. Photodiagn Photodyn Ther. (2023) 41:103297. doi: 10.1016/j.pdpdt.2023.103297, PMID: 36682429

[18] Pieklarz B Gińdzieńska-Sieśkiewicz E Zawadzka I Bagrowska M Daniluk J Palewski M. Macular choroidal thickness, volume, and vascularity index in patients with systemic sclerosis. Graefes Arch Clin Exp Ophthalmol. (2023) 262:1475–87. doi: 10.1007/s00417-023-06342-4, PMID: 38133798 PMC11031445

[19] Pieklarz B Gińdzieńska-Sieśkiewicz E Zawadzka I Bagrowska M Daniluk J Sidorczuk P. Peripapillary choroidal vascularity index and thickness in patients with systemic sclerosis. Front Med (Lausanne). (2023) 10:1273438. doi: 10.3389/fmed.2023.1273438, PMID: 37915331 PMC10617027

[20] Hayreh SS. In vivo choroidal circulation and its watershed zones. Eye (Lond). (1990) 4:273–89. doi: 10.1038/eye.1990.39, PMID: 2199236

[21] Iovino C Pellegrini M Bernabei F Borrelli E Sacconi R Govetto A. Choroidal vascularity index: an in-depth analysis of this novel optical coherence tomography parameter. J Clin Med. (2020) 9:595. doi: 10.3390/jcm9020595, PMID: 32098215 PMC7074450

[22] Kozikowska M Luboń W Kucharz E Mrukwa-Kominek E. Ocular manifestations in patients with systemic sclerosis. Reumatologia. (2020) 58:401–6. doi: 10.5114/reum.2020.102004, PMID: 33456083 PMC7792544

[23] Kim MS Lim HB Lee WH Kim KM Nam KY Kim JY. Wide-field swept-source optical coherence tomography analysis of interocular symmetry of choroidal thickness in healthy young individuals. Invest Ophthalmol Vis Sci. (2021) 62:5. doi: 10.1167/iovs.62.3.5, PMID: 33656554 PMC7938000

[24] Chen FK Yeoh J Rahman W Patel PJ Tufail A da Cruz L. Topographic variation and interocular symmetry of macular choroidal thickness using enhanced depth imaging optical coherence tomography. Invest Ophthalmol Vis Sci. (2012) 53:975–85. doi: 10.1167/iovs.11-8771, PMID: 22232433

[25] Yang M Wang W Xu Q Tan S Wei S. Interocular symmetry of the peripapillary choroidal thickness and retinal nerve fibre layer thickness in healthy adults with isometropia. BMC Ophthalmol. (2016) 16:182. doi: 10.1186/s12886-016-0361-7, PMID: 27756260 PMC5069918

[26] Al-Haddad C El Chaar L Antonios R El-Dairi M Noureddin B. Interocular symmetry in macular choroidal thickness in children. J Ophthalmol. (2014) 2014:472391. doi: 10.1155/2014/472391 25525509 PMC4265376

[27] Lu J Zhou H Shi Y Choe J Shen M Wang L. Interocular asymmetry of choroidal thickness and vascularity index measurements in normal eyes assessed by swept-source optical coherence tomography. Quant Imaging Med Surg. (2022) 12:781–95. doi: 10.21037/qims-21-813, PMID: 34993118 PMC8666769

[28] Goud A Singh SR Sahoo NK Rasheed MA Vupparaboina KK Ankireddy S. New insights on choroidal vascularity: a comprehensive topographic approach. Invest Ophthalmol Vis Sci. (2019) 60:3563–9. doi: 10.1167/iovs.18-26381, PMID: 31419299

[29] van den Hoogen F Khanna D Fransen J Johnson SR Baron M Tyndall A. 2013 classification criteria for systemic sclerosis: an American college of rheumatology/European league against rheumatism collaborative initiative. Ann Rheum Dis. (2013) 72:1747–55. doi: 10.1136/annrheumdis-2013-204424, PMID: 24092682

[30] Smith V Herrick AL Ingegnoli F Damjanov N de Angelis R Denton CP. Standardisation of nailfold capillaroscopy for the assessment of patients with Raynaud's phenomenon and systemic sclerosis. Autoimmun Rev. (2020) 19:102458. doi: 10.1016/j.autrev.2020.102458, PMID: 31927087

[31] Sonoda S Sakamoto T Yamashita T Shirasawa M Uchino E Terasaki H. Choroidal structure in normal eyes and after photodynamic therapy determined by binarization of optical coherence tomographic images. Invest Ophthalmol Vis Sci. (2014) 55:3893–9. doi: 10.1167/iovs.14-14447, PMID: 24894395

[32] Agrawal R Salman M Tan KA Karampelas M Sim DA Keane PA. Choroidal vascularity index (CVI) - a novel optical coherence tomography parameter for monitoring patients with panuveitis? PLoS One. (2016) 11:e0146344. doi: 10.1371/journal.pone.0146344, PMID: 26751702 PMC4713828

[33] Sigal IA Schuman JS Ishikawa H Kagemann L Wollstein G. A problem of proportions in OCT-based morphometry and a proposed solution. Invest Ophthalmol Vis Sci. (2016) 57:484–5. doi: 10.1167/iovs.15-18570, PMID: 26868751 PMC4758297

[34] Early Treatment Diabetic Retinopathy Study Research Group. Grading diabetic retinopathy from stereoscopic color fundus photographs--an extension of the modified Airlie House classification. ETDRS report number 10. Ophthalmology. (1991) 98:786–806. doi: 10.1016/S0161-6420(13)38012-9 2062513

[35] Koo TK Li MY. A guideline of selecting and reporting intraclass correlation coefficients for reliability research. J Chiropr Med. (2016) 15:155–63. doi: 10.1016/j.jcm.2016.02.012, PMID: 27330520 PMC4913118

[36] Armstrong RA. Statistical guidelines for the analysis of data obtained from one or both eyes. Ophthalmic Physiol Opt. (2013) 33:7–14. doi: 10.1111/opo.12009, PMID: 23252852

[37] Chan YH. Biostatistics 104: correlational analysis. Singapore Med J. (2003) 44:614–9. PMID: 14770254

[38] Pietrowska K Dmuchowska DA Godlewski A Grochowski ET Wojnar M Gosk W. Extent of interocular (a)symmetry based on the metabolomic profile of human aqueous humor. Front Mol Biosci. (2023) 10:1166182. doi: 10.3389/fmolb.2023.1166182, PMID: 37065449 PMC10090416

[39] Cutolo CA Cere A Toma P Cannavacciuolo T Toma C Balito S. Peripheral and ocular microvascular alterations in systemic sclerosis: observations from capillaroscopic assessments, perfusion peripheral analysis, and optical coherence tomography angiography. Rheumatol Int. (2024) 44:107–18. doi: 10.1007/s00296-023-05495-z, PMID: 37978075 PMC10766778

[40] Carnevali A Giannaccare G Gatti V Battaglia C Randazzo G Yu AC. Retinal microcirculation abnormalities in patients with systemic sclerosis: an explorative optical coherence tomography angiography study. Rheumatology (Oxford). (2021) 60:5827–32. doi: 10.1093/rheumatology/keab258, PMID: 33715001

[41] Hysa E Cutolo CA Gotelli E Paolino S Cimmino MA Pacini G. Ocular microvascular damage in autoimmune rheumatic diseases: the pathophysiological role of the immune system. Autoimmun Rev. (2021) 20:102796. doi: 10.1016/j.autrev.2021.102796, PMID: 33722750

[42] Maclean H Guthrie W. Retinopathy in scleroderma. Trans Ophthalmol Soc U K. (1962) 1970:209–20. 5276652

[43] Grennan DM Forrester J. Involvement of the eye in SLE and scleroderma. A study using fluorescein angiography in addition to clinical ophthalmic assessment. Ann Rheum Dis. (1977) 36:152–6. doi: 10.1136/ard.36.2.152, PMID: 856066 PMC1006650

[44] Serup L Serup J Hagdrup H. Fundus fluorescein angiography in generalized scleroderma. Ophthalmic Res. (1987) 19:303–8. doi: 10.1159/000265512, PMID: 3438051

[45] Kraus A Guerra-Bautista G Espinoza G Barojas E Quiroz-Mercado H Sanchez-Echeverri G. Defects of the retinal pigment epithelium in scleroderma. Br J Rheumatol. (1991) 30:112–4. doi: 10.1093/rheumatology/30.2.112, PMID: 2012937

[46] Ingegnoli F Gualtierotti R Pierro L del Turco C Miserocchi E Schioppo T. Choroidal impairment and macular thinning in patients with systemic sclerosis: the acute study. Microvasc Res. (2015) 97:31–6. doi: 10.1016/j.mvr.2014.08.008, PMID: 25262916

[47] Colaci M Zanoli L Lo Gullo A Sambataro D Sambataro G Aprile ML. The impaired elasticity of large arteries in systemic sclerosis patients. J Clin Med. (2022) 11:3256. doi: 10.3390/jcm11123256, PMID: 35743327 PMC9224949

[48] Esen E Tas DA Sizmaz S Turk I Unal I Demircan N. Evaluating choroidal characteristics in systemic sclerosis using enhanced depth imaging optical coherence tomography. Ocul Immunol Inflamm. (2017) 25:356–62. doi: 10.3109/09273948.2015.1129424, PMID: 26902374

[49] Coşkun E Zengin O Kenan S Kimyon G Erdogan Er K Okumus S. Evaluation of choroidal thickness in patients with scleroderma. Eye (Lond). (2016) 30:588–92. doi: 10.1038/eye.2015.287 26795407 PMC5108546

[50] Dimachkie MM Barohn RJ. Idiopathic inflammatory myopathies. Semin Neurol. (2012) 32:227–36. doi: 10.1055/s-0032-1329201, PMID: 23117947 PMC3534736

[51] Schanz S Henes J Ulmer A Kötter I Fierlbeck G Claussen CD. Magnetic resonance imaging findings in patients with systemic scleroderma and musculoskeletal symptoms. Eur Radiol. (2013) 23:212–21. doi: 10.1007/s00330-012-2584-1, PMID: 22843057

[52] Nitsche A. Raynaud, digital ulcers and calcinosis in scleroderma. Reumatol Clin. (2012) 8:270–7. doi: 10.1016/j.reuma.2012.02.006, PMID: 22835924

[53] Shenavandeh S Habibi S Habibi Y Nazarinia M. Mechanic hands: clinical and capillaroscopy manifestations of patients with connective tissue diseases presented with and without mechanic hands. Clin Rheumatol. (2019) 38:2309–18. doi: 10.1007/s10067-018-04422-z, PMID: 30635856

[54] Emre S Kaykçoğlu Ö Ateş H Çnar E İnceoğlu N Yargucu F. Corneal hysteresis, corneal resistance factor, and intraocular pressure measurement in patients with scleroderma using the reichert ocular response analyzer. Cornea. (2010) 29:628–31. doi: 10.1097/ICO.0b013e3181c3306a, PMID: 20458219

[55] Yasukawa T Hanyuda A Yamagishi K Yuki K Uchino M Ozawa Y. Relationship between blood pressure and intraocular pressure in the JPHC-NEXT eye study. Sci Rep. (2022) 12:17493. doi: 10.1038/s41598-022-22301-1, PMID: 36261671 PMC9582013

[56] Parajuli S Shrestha P Shrestha JK Sharma S. Comparison of intraocular pressure among individuals with systemic hypertension and those with normal blood pressure. Nepal J Ophthalmol. (2021) 13:137–44. doi: 10.3126/nepjoph.v13i2.33917, PMID: 35996780

[57] Tsai AS Aung T Yip W Wong TY Cheung CY. Relationship of intraocular pressure with central aortic systolic pressure. Curr Eye Res. (2016) 41:377–82. doi: 10.3109/02713683.2015.1030506, PMID: 25942602

