# Supplementary material for: Assessment of interocular symmetry of choroidal vascularity index and thickness in patients with systemic sclerosis: a prospective study
Source: Front Med (Lausanne). 2025 Jan 16;11:1513679. doi: 10.3389/fmed.2024.1513679 (PMC11781297; doi:10.3389/fmed.2024.1513679)
Supplement: Supplementary file 1 [file Data_Sheet_1.docx]

**Supplementary Material**

**Supplementary Table 1.** Signed and absolute interocular differences for choroidal parameters in SSc patients

|  |  |  | |  | |  | |  |
| --- | --- | --- | --- | --- | --- | --- | --- | --- |
|  | Signed interocular differences (left eye - right eye) | | | Absolute interocular differences | | | | |
|  | Mean (SD) | | Normal 95% limits (2.5% to 97.5% title) | Mean (SD) | Median (min;max) | | Normal 95% limits (95% tile) | |
| mTCA, µm^2^ | -8 229.46 (48 070.53) | | -81 069.68 to 76 074.20 | 37 773.25 (30 022.41) | 35 309.75 (266.50;114 692.50) | | 86 390.03 | |
| mLA, µm^2^ | -4 233.64 (31 818.25) | | -59 085.85 to 50 835.88 | 25 596.00 (18 750.15) | 22 132.75 (1 619.50;64 936.00) | | 61 227.15 | |
| mSA, µm^2^ | -3 995.82 (19 168.72) | | -39 978.73 to 20 822.88 | 13 698.00 (13 762.75) | 9 262.25 (175.00;49 756.50) | | 39 978.73 | |
| mCVI, % | 0.08 (2.57) | | -4.06 to 4.91 | 1.85 (1.75) | 1.16 (0.14;6.33) | | 5.69 | |
| Central macular choroidal thickness, µm | -10.04 (47.33) | | -91.10 to 57.20 | 39.00 (27.67) | 32.00 (1.00;106.00) | | 91.10 | |
| SFCT, µm | -15.56 (47.16) | | -99.20 to 45.30 | 40.89 (27.19) | 34.00 (8.00;118.00) | | 99.20 | |
| Central macular choroidal volume, µm^3^ | -0.01 (0.04) | | -0.07 to 0.04 | 0.03 (0.02) | 0.03 (0.00;0.09) | | 0.07 | |
| Total choroidal volume, µm^3^ | 0.00 (0.70) | | -0.93 to 1.38 | 0.51 (0.48) | 0.31 (0.00;1.83) | | 1.40 | |
| pTCA, µm^2^ | 64 928.32 (270 754.86) | | -376 684.95 to 460 153.70 | 221 105.82 (164 607.66) | 162 854.50 (407.00;632 978.50) | | 524 922.30 | |
| pLA, µm^2^ | 53 470.92 (177 498.45) | | -192 204.80 to 333 153.23 | 149 534.08 (106 451.57) | 122 849.50 (3 945.50;436 064.00) | | 342 576.45 | |
| pSA, µm^2^ | 11 457.40 (105 842.07) | | -185 630.75 to 167 582.98 | 83 114.67 (64 746.33) | 63 180.25 (923.50;210 433.00) | | 195 594.20 | |
| pCVI, % | 0.64 (1.62) | | -1.50 to 2.69 | 1.37 (1.06) | 1.08 (0.02;4.65) | | 2.92 | |
| pCT Global, µm | 4.33 (22.19) | | -28.80 to 37.20 | 17.79 (13.62) | 16.00 (2.00;52.00) | | 42.80 | |
| pCT S, µm | -3.06 (26.85) | | -37.00 to 43.40 | 20.94 (16.69) | 14.00 (1.00;68.00) | | 53.20 | |
| pCT I, µm | 7.52 (30.61) | | -29.80 to 57.60 | 23.45 (20.68) | 19.00 (1.00;87.00) | | 62.40 | |
| pCT T, µm | 7.94 (26.85) | | -31.80 to 43.00 | 23.27 (15.08) | 20.00 (2.00;59.00) | | 48.60 | |
| pCT N, µm | 4.79 (33.83) | | -43.00 to 60.00 | 28.97 (17.40) | 29.00 (1.00;65.00) | | 60.00 | |

Abbreviations: CT, choroidal thickness; CVI, choroidal vascularity index; I, inferior; LA, luminal area; m, macular; N, nasal; p, peripapillary; S, superior; SA, stromal area; SD, standard deviation; SFCT, subfoveal choroidal thickness; T, temporal; TCA, total choroidal area

**Supplementary Table 2**. Signed and absolute interocular differences for choroidal parameters in controls

|  | Signed interocular differences (left eye - right eye) | | Absolute interocular differences | | |
| --- | --- | --- | --- | --- | --- |
|  | Mean (SD) | Normal 95% limits (2.5% to 97.5% title) | Mean (SD) | Median (min;max) | Normal 95% limits (95% tile) |
| mTCA, µm^2^ | -1 956.25 (60 383.58) | -108 044.60 to 85 632.85 | 48 168.86 (35 597.18) | 40 893.75 (1 786.00;136 704.50) | 118 967.53 |
| mLA, µm^2^ | -339.03 (39 982.71) | -64 924.58 to 60 121.80 | 32 321.95 (22 930.14) | 25 907.50 (1 411.50;88 103.00) | 70 525.18 |
| mSA, µm^2^ | -1 617.22 (23 066.75) | -33 641.30 to 28 849.85 | 17 962.38 (14 261.62) | 15 991.75 (771.50;62 775.00) | 45 259.13 |
| mCVI, % | 0.55 (2.65) | -3.60 to 4.59 | 2.19 (1.56) | 1.92 (0.09;7.44) | 4.59 |
| Central macular choroidal thickness, µm | 6.46 (57.90) | -95.70 to 89.20 | 46.51 (34.28) | 41.00 (2.00;162.00) | 95.70 |
| SFCT, µm | 4.08 (76.00) | -119.10 to 115.60 | 60.28 (45.43) | 43.00 (1.00;179.00) | 131.70 |
| Central macular choroidal volume, µm^3^ | 0.00 (0.05) | -0.07 to 0.07 | 0.04 (0.03) | 0.03 (0.00;0.14) | 0.07 |
| Total choroidal volume, µm^3^ | -0.15 (0.93) | -1.62 to 1.50 | 0.74 (0.57) | 0.49 (0.05;2.07) | 1.68 |
| pTCA, µm^2^ | -88 178.04 (287 176.55) | -565 195.55 to 276 001.25 | 232 201.99 (187 469.56) | 192 370.00 (1 024.50;783 140.50) | 565 195.55 |
| pLA, µm^2^ | -63 845.41 (195 039.47) | -380 674.48 to 195 370.00 | 149 336.16 (139 097.21) | 107 936.75 (2 597.00;586 889.00) | 380 674.47 |
| pSA, µm^2^ | -24 332.63 (114 483.68) | -200 294.98 to 118 454.28 | 92 529.95 (70 231.37) | 91 812.50 (2 105.00;289 518.00) | 200 294.98 |
| pCVI, % | -0.21 (2.12) | -3.31 to 3.15 | 1.72 (1.23) | 1.61 (0.03;4.51) | 3.99 |
| pCT Global, µm | -7.35 (26.96) | -49.45 to 30.05 | 20.20 (19.07) | 18.50 (0.00;95.00) | 49.45 |
| pCT S, µm | -13.83 (36.37) | -61.20 to 42.40 | 29.03 (25.58) | 27.50 (0.00;142.00) | 61.20 |
| pCT I, µm | -3.10 (31.64) | -47.25 to 42.10 | 25.15 (19.04) | 21.00 (0.00;87.00) | 52.40 |
| pCT T, µm | -6.58 (34.24) | -56.70 to 33.00 | 24.78 (24.24) | 19.50 (1.00;131.00) | 56.70 |
| pCT N, µm | -6.18 (36.03) | -67.75 to 38.75 | 26.53 (24.82) | 17.50 (2.00;94.00) | 73.45 |

Abbreviations: CT, choroidal thickness; CVI, choroidal vascularity index; I, inferior; LA, luminal area; m, macular; N, nasal; p, peripapillary; S, superior; SA, stromal area; SD, standard deviation; SFCT, subfoveal choroidal thickness; T, temporal; TCA, total choroidal area

**Supplementary Table 3.1**. Values of the choroidal parameters in controls by sex

| Group | Variable | Mean ±SD left eyes | Mean ±SD right eyes | Median (Q1;Q3) left eyes | Median (Q1;Q3) right eyes | Relative mean difference (%) between left and right eyes |
| --- | --- | --- | --- | --- | --- | --- |
| Control group - females | mTCA, µm^2^ | 361 666.70±78 918.75 | 374 495.07±90 559.22 | 359 821.25 (304 275.38;397 873.63) | 377 456.25 (336 002.75;411 960.25) | -3.49 |
|  | mLA, µm^2^ | 241 621.45±47 219.21 | 249 344.02±59 297.44 | 242 195.25 (203 872.13;260 418.88) | 263 840.75 (215 639.38;272 242.38) | -3.15 |
|  | mSA, µm^2^ | 120 045.25±33 284.96 | 125 151.05±33 665.58 | 120 723.50 (98 892.50;138 235.25) | 119 443.25 (104 500.63;148 290.75) | -4.16 |
|  | mCVI, % | 67.17±2.94 | 66.65±2.63 | 66.08 (65.36;69.54) | 65.93 (64.76;67.51) | 0.78 |
|  | Central macular choroidal thickness, µm | 327.95±61.48 | 314.73±78.42 | 326.00 (292.25;360.75) | 311.00 (261.25;368.00) | 4.12 |
|  | SFCT, µm | 322.73±63.68 | 317.23±92.48 | 313.50 (281.50;362.00) | 284.00 (259.50;355.50) | 1.72 |
|  | Central macular choroidal volume, µm^3^ | 0.26±0.05 | 0.25±0.06 | 0.26 (0.23;0.29) | 0.24 (0.20;0.29) | 3.98 |
|  | Total choroidal volume, µm^3^ | 7.98±1.52 | 8.08±1.76 | 8.04 (7.08;8.67) | 7.74 (6.90;8.87) | -1.22 |
|  | pTCA, µm^2^ | 2 447 802.48±537 183.66 | 2 549 496.66±651 378.48 | 2 363 216.00 (2 143 531.13;2 755 190.75) | 2 579 502.25 (2 020 839.63;2 926 578.25) | -4.07 |
|  | pLA, µm^2^ | 1 618 521.82±365 902.75 | 1 696 447.02±441 832.01 | 1 568 973.00 (1 391 251.25;1 838 766.63) | 1 695 079.75 (1 344 914.38;1 983 695.63) | -4.70 |
|  | pSA, µm^2^ | 829 280.66±182 123.85 | 853 049.64±220 143.45 | 853 966.25 (693 723.88;911 106.25) | 801 502.50 (682 919.38;963 885.25) | -2.83 |
|  | pCVI, % | 66.08±2.25 | 66.44±2.20 | 66.24 (64.71;66.82) | 66.16 (65.10;68.00) | -0.55 |
|  | pCT Global, µm | 195.73±44.32 | 206.55±58.21 | 188.00 (169.00;207.75) | 202.50 (160.00;234.50) | -5.38 |
|  | pCT S, µm | 209.00±50.50 | 224.45±61.52 | 200.50 (170.75;232.75) | 216.00 (177.75;259.50) | -7.13 |
|  | pCT I, µm | 170.82±45.32 | 176.23±61.88 | 165.50 (149.25;186.00) | 171.00 (124.25;210.50) | -3.12 |
|  | pCT T, µm | 206.18±45.71 | 218.86±63.30 | 202.00 (177.00;227.00) | 215.00 (180.75;241.25) | -5.97 |
|  | pCT N, µm | 197.05±47.44 | 207.09±61.13 | 192.50 (166.75;221.25) | 204.50 (161.75;255.00) | -4.97 |
| Control group - males | mTCA, µm^2^ | 356 517.22±85 720.94 | 343 524.31±105 988.07 | 349 346.50 (301 631.00;437 030.63) | 355 772.50 (264 702.13;428 679.25) | 3.71 |
|  | mLA, µm^2^ | 233 382.84±52 713.82 | 223 569.50±68 044.88 | 234 166.00 (198 942.00;275 758.00) | 243 849.75 (178 145.25;271 059.88) | 4.30 |
|  | mSA, µm^2^ | 123 134.38±34 725.98 | 119 954.81±39 191.28 | 115 215.00 (98 729.38;157 739.13) | 117 940.50 (87 867.75;154 047.25) | 2.62 |
|  | mCVI, % | 65.75±3.00 | 65.15±2.41 | 64.97 (63.73;67.27) | 65.20 (63.28;66.89) | 0.91 |
|  | Central macular choroidal thickness, µm | 315.06±86.50 | 314.17±107.35 | 320.00 (260.00;401.00) | 316.00 (238.25;398.50) | 0.28 |
|  | SFCT, µm | 319.94±88.32 | 313.67±114.94 | 301.00 (262.00;404.00) | 310.50 (228.50;398.75) | 1.98 |
|  | Central macular choroidal volume, µm^3^ | 0.25±0.07 | 0.25±0.09 | 0.25 (0.21;0.32) | 0.25 (0.19;0.32) | -0.49 |
|  | Total choroidal volume, µm^3^ | 7.70±1.91 | 7.85±2.02 | 7.55 (6.60;9.30) | 8.24 (6.38;9.50) | -1.88 |
|  | pTCA, µm^2^ | 2 574 043.31±675 876.71 | 2 645 701.61±703 431.46 | 2 437 899.75 (2 247 909.50;3 092 214.75) | 2 660 660.50 (2 021 432.75;3 116 197.38) | -2.75 |
|  | pLA, µm^2^ | 1 681 038.83±469 189.43 | 1 727 675.61±476 772.54 | 1 607 316.00 (1 415 328.38;2 075 878.00) | 1 777 573.50 (1 274 669.75;2 044 630.13) | -2.74 |
|  | pSA, µm^2^ | 893 004.47±213 048.83 | 918 026.00±232 494.59 | 858 835.50 (780 608.75;1 052 427.63) | 906 932.50 (721 332.00;1 062 972.13) | -2.76 |
|  | pCVI, % | 65.07±1.87 | 65.09±1.85 | 64.82 (64.09;66.25) | 65.20 (64.44;65.88) | -0.04 |
|  | pCT Global, µm | 203.83±56.23 | 206.94±61.02 | 199.50 (163.50;252.75) | 199.50 (158.50;241.50) | -1.51 |
|  | pCT S, µm | 214.17±60.62 | 226.00±64.58 | 214.50 (157.50;259.50) | 215.00 (174.75;273.50) | -5.38 |
|  | pCT I, µm | 182.06±55.05 | 182.33±60.57 | 177.00 (138.00;222.25) | 169.50 (137.25;211.25) | -0.15 |
|  | pCT T, µm | 214.83±59.07 | 213.94±60.83 | 213.50 (173.00;257.75) | 212.50 (171.50;260.25) | 0.41 |
|  | pCT N, µm | 204.39±60.28 | 205.83±71.99 | 185.00 (166.00;254.25) | 189.50 (157.25;243.50) | -0.70 |

Abbreviations: CT, choroidal thickness; CVI, choroidal vascularity index; I, inferior; LA, luminal area; m, macular; N, nasal; p, peripapillary; Q1, quartile 1; Q3, quartile 3; S, superior; SA, stromal area; SD, standard deviation; SFCT, subfoveal choroidal thickness; T, temporal; TCA, total choroidal area

Relative mean difference calculated as per formula: (left eye - right eye) / average from both eyes * 100

**Supplementary Table 3.2**. Comparison of the choroidal parameters in fellow eyes in controls by sex

| Group | Variable | ICC between both eyes | 95% CI for ICC | r between both eyes | p value for r | p-value of Wilcoxon test (w/o correction)^a^ | p-value of Wilcoxon test (B-H correction)^a^ |
| --- | --- | --- | --- | --- | --- | --- | --- |
| Control group - females | mTCA | 0.775 | 0.537-0.899 | 0.83 | **<0.001** | 0.290 | 0.591 |
|  | mLA | 0.741 | 0.477-0.883 | 0.68 | **0.001** | 0.337 | 0.591 |
|  | mSA | 0.798 | 0.580-0.910 | 0.84 | **<0.001** | 0.248 | 0.591 |
|  | mCVI | 0.532 | 0.156-0.774 | 0.40 | 0.066 | 0.443 | 0.591 |
|  | Central macular choroidal thickness | 0.679 | 0.377-0.852 | 0.62 | **0.002** | 0.372 | 0.591 |
|  | SFCT | 0.509 | 0.113-0.764 | 0.50 | **0.018** | 0.685 | 0.702 |
|  | Central macular choroidal volume | 0.685 | 0.386-0.855 | 0.62 | **0.002** | 0.340 | 0.591 |
|  | Total choroidal volume | 0.799 | 0.576-0.912 | 0.65 | **0.001** | 0.702 | 0.702 |
|  | pTCA | 0.869 | 0.711-0.943 | 0.88 | **<0.001** | 0.235 | 0.423 |
|  | pLA | 0.866 | 0.699-0.942 | 0.90 | **<0.001** | 0.129 | 0.387 |
|  | pSA | 0.834 | 0.646-0.927 | 0.84 | **<0.001** | 0.545 | 0.592 |
|  | pCVI | 0.578 | 0.219-0.800 | 0.50 | **0.018** | 0.503 | 0.592 |
|  | pCT Global | 0.81 | 0.596-0.916 | 0.86 | **<0.001** | 0.175 | 0.394 |
|  | pCT S | 0.724 | 0.445-0.875 | 0.81 | **<0.001** | 0.118 | 0.387 |
|  | pCT I | 0.763 | 0.512-0.894 | 0.73 | **<0.001** | 0.532 | 0.592 |
|  | pCT T | 0.817 | 0.603-0.920 | 0.83 | **<0.001** | 0.105 | 0.387 |
|  | pCT N | 0.772 | 0.533-0.898 | 0.81 | **<0.001** | 0.592 | 0.592 |
| Control group - males | mTCA | 0.786 | 0.495-0.919 | 0.67 | **0.005** | 0.464 | 0.677 |
|  | mLA | 0.777 | 0.481-0.915 | 0.69 | **0.004** | 0.348 | 0.677 |
|  | mSA | 0.774 | 0.465-0.915 | 0.76 | **0.001** | 0.433 | 0.677 |
|  | mCVI | 0.526 | 0.065-0.803 | 0.59 | **0.018** | 0.495 | 0.677 |
|  | Central macular choroidal thickness | 0.821 | 0.571-0.932 | 0.78 | **<0.001** | 0.722 | 0.825 |
|  | SFCT | 0.759 | 0.446-0.906 | 0.78 | **<0.001** | 0.507 | 0.677 |
|  | Central macular choroidal volume | 0.808 | 0.545-0.926 | 0.80 | **<0.001** | 0.877 | 0.877 |
|  | Total choroidal volume | 0.927 | 0.815-0.973 | 0.93 | **<0.001** | 0.407 | 0.677 |
|  | pTCA | 0.915 | 0.792-0.967 | 0.92 | **<0.001** | 0.442 | 0.708 |
|  | pLA | 0.918 | 0.799-0.968 | 0.92 | **<0.001** | 0.417 | 0.708 |
|  | pSA | 0.868 | 0.687-0.948 | 0.90 | **<0.001** | 0.417 | 0.708 |
|  | pCVI | 0.282 | -0.227-0.659 | 0.28 | **0.260** | 0.551 | 0.708 |
|  | pCT Global | 0.937 | 0.842-0.976 | 0.94 | **<0.001** | 0.777 | 0.777 |
|  | pCT S | 0.861 | 0.668-0.946 | 0.89 | **<0.001** | *0.119* | 0.708 |
|  | pCT I | 0.926 | 0.813-0.972 | 0.92 | **<0.001** | 0.744 | 0.777 |
|  | pCT T | 0.825 | 0.588-0.931 | 0.77 | **<0.001** | 0.170 | 0.708 |
|  | pCT N | 0.861 | 0.665-0.946 | 0.84 | **<0.001** | 0.472 | 0.708 |

Abbreviations: CI, confidence interval; CT, choroidal thickness; CVI, choroidal vascularity index; I, inferior; ICC, intraclass correlation coefficient; LA, luminal area; m, macular; N, nasal; p, peripapillary; r, Spearman correlation coefficient; S, superior; SA, stromal area; SFCT, subfoveal choroidal thickness; T, temporal; TCA, total choroidal area

p<0.05 in bold font

Wilcoxon test comparing average level between left eyes and right eyes

a p-value after Benjamini-Hochberg correction for multiple comparisons. The correction was made separately for macular (8 comparisons) and peripapillary (9 comparisons) choroidal parameters.

**Supplementary Table 3.3.** Values of the choroidal parameters in SSc patients by sex

| Group | Variable | Mean ±SD left eyes | Mean ±SD right eyes | Median (Q1;Q3) left eyes | Median (Q1;Q3) right eyes | Relative mean difference (%) between left and right eyes |
| --- | --- | --- | --- | --- | --- | --- |
| SSc - females | mTCA, µm^2^ | 309 822.12±60 672.48 | 328 769.89±67 869.28 | 313 655.50 (273 208.50;350 419.00) | 320 190.50 (297 889.50;377 189.00) | -5.93 |
|  | mLA, µm^2^ | 207 286.38±42 995.89 | 219 689.89±43 023.08 | 212 730.50 (185 300.00;234 907.50) | 217 020.50 (199 498.25;248 516.25) | -5.81 |
|  | mSA, µm^2^ | 102 535.74±19 353.38 | 109 080.00±27 039.81 | 104 590.00 (91 603.50;114 446.00) | 104 635.00 (89 962.75;126 381.25) | -6.19 |
|  | mCVI, % | 66.75±2.50 | 66.99±2.94 | 66.65 (65.79;67.84) | 66.80 (65.07;68.47) | -0.37 |
|  | Central macular choroidal thickness, µm | 275.10±58.44 | 294.05±72.08 | 281.50 (237.00;295.75) | 283.00 (256.75;357.25) | -6.66 |
|  | SFCT, µm | 275.20±63.11 | 300.55±69.61 | 278.00 (234.75;310.25) | 295.00 (241.50;356.25) | -8.80 |
|  | Central macular choroidal volume, µm^3^ | 0.22±0.04 | 0.23±0.06 | 0.22 (0.19;0.23) | 0.22 (0.20;0.28) | -7.10 |
|  | Total choroidal volume, µm^3^ | 7.21±1.24 | 7.39±1.65 | 7.45 (6.38;7.72) | 7.44 (6.20;8.29) | -2.38 |
|  | pTCA, µm^2^ | 2 495 996.10±718 435.29 | 2 464 476.59±763 270.65 | 2 458 968.00 (2 018 275.38;3 110 452.63) | 2 405 307.75 (1 916 738.38;2 918 891.25) | 1.27 |
|  | pLA, µm^2^ | 1 611 799.06±481 297.56 | 1 584 034.02±515 652.90 | 1 553 999.00 (1 248 859.13;2 006 678.75) | 1 546 367.75 (1 196 740.00;1 957 321.25) | 1.74 |
|  | pSA, µm^2^ | 884 197.04±242 934.98 | 880 442.57±254 186.14 | 928 595.75 (709 412.25;1 060 444.25) | 844 418.25 (725 241.50;1 008 807.13) | 0.43 |
|  | pCVI, % | 64.41±1.80 | 63.91±2.30 | 64.26 (63.11;65.69) | 64.36 (62.20;65.22) | 0.77 |
|  | pCT Global, µm | 198.63±65.60 | 193.13±60.16 | 190.00 (157.00;246.75) | 184.00 (151.75;235.50) | 2.81 |
|  | pCT S, µm | 205.00±62.74 | 208.63±66.09 | 210.50 (172.50;254.25) | 194.50 (157.25;260.50) | -1.75 |
|  | pCT I, µm | 183.21±68.47 | 172.17±56.66 | 178.00 (124.50;231.00) | 160.50 (133.50;212.00) | 6.21 |
|  | pCT T, µm | 207.58±82.92 | 199.67±69.51 | 191.00 (145.25;271.75) | 184.00 (155.50;250.00) | 3.89 |
|  | pCT N, µm | 198.88±61.68 | 192.50±58.61 | 208.00 (162.50;237.00) | 194.00 (152.50;231.00) | 3.26 |
| SSc - males | mTCA, µm^2^ | 337 848.50±70 885.25 | 330 226.86±62 154.70 | 324 922.00 (289 491.00;384 156.25) | 308 368.00 (282 957.00;373 965.75) | 2.28 |
|  | mLA, µm^2^ | 232 633.43±47 306.69 | 223 718.07±37 509.96 | 219 334.00 (200 475.00;264 724.75) | 207 831.00 (198 599.00;249 257.25) | 3.91 |
|  | mSA, µm^2^ | 105 215.07±24 051.53 | 106 508.79±26 313.88 | 102 250.00 (88 626.25;121 490.25) | 100 537.00 (88 633.25;122 497.25) | -1.22 |
|  | mCVI, % | 68.95±1.35 | 67.99±2.52 | 68.32 (68.06;70.10) | 67.49 (67.13;69.71) | 1.40 |
|  | Central macular choroidal thickness, µm | 295.67±69.67 | 284.88±61.36 | 288.00 (260.00;368.00) | 289.00 (269.00;319.25) | 3.72 |
|  | SFCT, µm | 290.89±66.64 | 289.00±62.13 | 282.00 (241.00;354.00) | 297.50 (255.75;326.50) | 0.65 |
|  | Central macular choroidal volume, µm^3^ | 0.23±0.06 | 0.22±0.05 | 0.23 (0.21;0.29) | 0.23 (0.21;0.25) | 4.67 |
|  | Total choroidal volume, µm^3^ | 7.60±1.50 | 7.28±1.74 | 7.22 (6.79;8.90) | 7.11 (6.55;8.33) | 4.26 |
|  | pTCA, µm^2^ | 2 288 428.50±593 738.23 | 2 085 900.50±442 079.47 | 2 175 458.50 (1 859 888.00;2 586 989.00) | 1 914 537.50 (1 783 430.63;2 363 751.88) | 9.26 |
|  | pLA, µm^2^ | 1 486 653.72±395 879.11 | 1 339 144.44±316 302.26 | 1 425 379.50 (1 224 166.50;1 668 228.50) | 1 211 021.00 (1 136 835.75;1 516 242.63) | 10.44 |
|  | pSA, µm^2^ | 801 774.78±201 103.55 | 746 756.06±133 088.63 | 750 079.00 (649 396.00;918 760.50) | 714 834.50 (631 555.13;838 317.00) | 7.11 |
|  | pCVI, % | 64.88±1.34 | 63.99±1.95 | 64.93 (64.48;65.75) | 63.74 (62.42;65.00) | 1.39 |
|  | pCT Global, µm | 180.11±53.30 | 178.89±51.49 | 182.00 (147.00;221.00) | 156.00 (142.00;200.00) | 0.68 |
|  | pCT S, µm | 185.67±53.37 | 187.22±53.80 | 184.00 (156.00;206.00) | 176.00 (140.00;207.00) | -0.83 |
|  | pCT I, µm | 163.22±66.24 | 165.11±52.72 | 181.00 (103.00;222.00) | 141.00 (132.00;195.00) | -1.15 |
|  | pCT T, µm | 192.89±50.68 | 184.89±62.54 | 196.00 (163.00;229.00) | 166.00 (139.00;203.00) | 4.24 |
|  | pCT N, µm | 178.44±51.55 | 177.89±44.29 | 190.00 (138.00;219.00) | 169.00 (150.00;194.00) | 0.31 |

Abbreviations: CT, choroidal thickness; CVI, choroidal vascularity index; I, inferior; LA, luminal area; m, macular; N, nasal; p, peripapillary; Q1, quartile 1; Q3, quartile 3; S, superior; SA, stromal area; SD, standard deviation; SFCT, subfoveal choroidal thickness; T, temporal; TCA, total choroidal area

Relative mean difference calculated as per formula: (left eye - right eye) / average from both eyes * 100

**Supplementary Table 3.4.** Comparison of the choroidal parameters in fellow eyes in SSc patients by sex

| Group | Variable | ICC between both eyes | 95% CI for ICC | r between both eyes | p value for r | p-value of Wilcoxon test (w/o correction)^a^ | p-value of Wilcoxon test (B-H correction)^a^ |
| --- | --- | --- | --- | --- | --- | --- | --- |
| SSc - females | mTCA | 0.718 | 0.433-0.874 | 0.57 | **0.008** | 0.216 | 0.366 |
|  | mLA | 0.702 | 0.406-0.866 | 0.54 | **0.013** | 0.229 | 0.366 |
|  | mSA | 0.712 | 0.423-0.871 | 0.66 | **0.001** | 0.562 | 0.642 |
|  | mCVI | 0.587 | 0.212-0.810 | 0.55 | **0.011** | 0.708 | 0.708 |
|  | Central macular choroidal thickness | 0.778 | 0.516-0.908 | 0.70 | **0.001** | 0.212 | 0.366 |
|  | SFCT | 0.766 | 0.478-0.904 | 0.76 | **<0.001** | 0.080 | 0.366 |
|  | Central macular choroidal volume | 0.752 | 0.466-0.896 | 0.73 | **<0.001** | 0.169 | 0.366 |
|  | Total choroidal volume | 0.898 | 0.756-0.960 | 0.83 | **<0.001** | 0.398 | 0.531 |
|  | pTCA | 0.937 | 0.856-0.973 | 0.94 | **<0.001** | 0.305 | 0.444 |
|  | pLA | 0.939 | 0.860-0.974 | 0.92 | **<0.001** | 0.276 | 0.444 |
|  | pSA | 0.913 | 0.802-0.963 | 0.91 | **<0.001** | 0.425 | 0.466 |
|  | pCVI | 0.622 | 0.293-0.822 | 0.70 | **<0.001** | 0.098 | 0.444 |
|  | pCT Global | 0.942 | 0.872-0.974 | 0.94 | **<0.001** | 0.259 | 0.444 |
|  | pCT S | 0.912 | 0.808-0.961 | 0.90 | **<0.001** | 0.345 | 0.444 |
|  | pCT I | 0.886 | 0.747-0.950 | 0.92 | **<0.001** | 0.137 | 0.444 |
|  | pCT T | 0.938 | 0.863-0.973 | 0.94 | **<0.001** | 0.153 | 0.444 |
|  | pCT N | 0.848 | 0.684-0.931 | 0.86 | **<0.001** | 0.466 | 0.466 |
| SSc - males | mTCA | 0.764 | 0.103-0.955 | 0.79 | **0.048** | 0.578 | >0.999 |
|  | mLA | 0.824 | 0.333-0.967 | 0.71 | 0.088 | 0.469 | >0.999 |
|  | mSA | 0.595 | 0.001-0.919 | 0.61 | 0.167 | >0.999 | >0.999 |
|  | mCVI | 0.209 | 0.001-0.796 | 0.46 | 0.302 | 0.578 | >0.999 |
|  | Central macular choroidal thickness | 0.611 | 0.001-0.910 | 0.69 | 0.069 | >0.999 | >0.999 |
|  | SFCT | 0.623 | 0.001-0.913 | 0.67 | 0.083 | >0.999 | >0.999 |
|  | Central macular choroidal volume | 0.612 | 0.001-0.910 | 0.60 | 0.114 | 0.945 | >0.999 |
|  | Total choroidal volume | 0.864 | 0.459-0.971 | 0.86 | **0.011** | >0.999 | >0.999 |
|  | pTCA | 0.826 | 0.393-0.962 | 0.74 | **0.046** | 0.461 | 0.830 |
|  | pLA | 0.842 | 0.444-0.966 | 0.81 | **0.022** | 0.383 | 0.830 |
|  | pSA | 0.768 | 0.189-0.949 | 0.74 | **0.046** | 0.461 | 0.830 |
|  | pCVI | 0.684 | 0.017-0.928 | 0.91 | **0.005** | **0.039** | 0.351 |
|  | pCT Global | 0.89 | 0.584-0.974 | 0.93 | **0.001** | 0.910 | >0.999 |
|  | pCT S | 0.886 | 0.570-0.973 | 0.82 | **0.011** | 0.678 | >0.999 |
|  | pCT I | 0.843 | 0.438-0.962 | 0.80 | **0.014** | >0.999 | >0.999 |
|  | pCT T | 0.867 | 0.542-0.968 | 0.89 | **0.001** | 0.407 | 0.830 |
|  | pCT N | 0.725 | 0.137-0.932 | 0.75 | **0.025** | >0.999 | >0.999 |

Abbreviations: CI, confidence interval; CT, choroidal thickness; CVI, choroidal vascularity index; I, inferior; ICC, intraclass correlation coefficient; LA, luminal area; m, macular; N, nasal; p, peripapillary; r, Spearman correlation coefficient; S, superior; SA, stromal area; SFCT, subfoveal choroidal thickness; T, temporal; TCA, total choroidal area

p<0.05 in bold font

Wilcoxon test comparing average level between left eyes and right eyes

a p-value after Benjamini-Hochberg correction for multiple comparisons. The correction was made separately for macular (8 comparisons) and peripapillary (9 comparisons) choroidal parameters.

**Supplementary Table 4.1**. Values of the choroidal parameters in SSc patients by SSc subtype

| Group | Variable | Mean ±SD left eyes | Mean ±SD right eyes | Median (Q1;Q3) left eyes | Median (Q1;Q3) right eyes | Relative mean difference (%) between left and right eyes |
| --- | --- | --- | --- | --- | --- | --- |
| SSc - limited | mTCA, µm^2^ | 326 522.61±68 689.96 | 350 127.40±76 429.12 | 333 983.50 (300 184.00;366 006.50) | 357 973.50 (300 054.38;406 185.75) | -6.98 |
|  | mLA, µm^2^ | 217 806.72±44 740.72 | 232 545.40±44 639.15 | 221 095.50 (198 344.50;242 908.50) | 232 879.50 (204 638.75;260 942.88) | -6.55 |
|  | mSA, µm^2^ | 108 715.89±25 306.81 | 117 582.00±32 796.58 | 115 511.50 (92 560.00;123 098.00) | 119 862.75 (94 294.88;146 083.25) | -7.84 |
|  | mCVI, % | 66.86±2.41 | 66.89±3.12 | 66.37 (65.79;67.13) | 66.56 (65.06;67.52) | -0.06 |
|  | Central macular choroidal thickness, µm | 271.00±60.91 | 303.00±90.64 | 271.50 (233.25;303.75) | 313.00 (257.00;357.25) | -11.15 |
|  | SFCT, µm | 274.00±61.11 | 312.70±84.63 | 267.50 (228.00;314.75) | 323.50 (265.50;382.50) | -13.19 |
|  | Central macular choroidal volume, µm^3^ | 0.21±0.05 | 0.24±0.07 | 0.21 (0.18;0.24) | 0.25 (0.20;0.28) | -12.03 |
|  | Total choroidal volume, µm^3^ | 7.04±1.23 | 7.24±1.72 | 7.26 (6.03;7.86) | 7.74 (6.20;8.29) | -2.80 |
|  | pTCA, µm^2^ | 2 436 534.77±696 868.38 | 2 271 886.40±560 431.34 | 2 474 622.00 (1 865 533.25;2 977 522.25) | 2 361 765.50 (1 796 642.13;2 724 789.63) | 6.99 |
|  | pLA, µm^2^ | 1 577 604.41±466 615.07 | 1 466 718.15±385 510.86 | 1 560 582.50 (1 185 021.00;1 952 417.75) | 1 546 367.75 (1 108 521.75;1 775 403.88) | 7.28 |
|  | pSA, µm^2^ | 858 930.36±235 437.65 | 805 168.25±178 145.62 | 927 206.50 (680 512.25;1 031 740.75) | 815 397.75 (688 120.38;949 385.75) | 6.46 |
|  | pCVI, % | 64.63±1.67 | 64.32±1.74 | 64.21 (63.99;65.57) | 64.76 (63.30;65.63) | 0.48 |
|  | pCT Global, µm | 185.55±61.95 | 179.36±43.80 | 189.00 (124.50;241.00) | 182.00 (142.00;218.50) | 3.39 |
|  | pCT S, µm | 187.09±58.23 | 193.27±55.29 | 181.00 (130.00;226.50) | 193.00 (143.50;239.50) | -3.25 |
|  | pCT I, µm | 173.73±54.62 | 160.27±37.20 | 182.00 (122.00;218.00) | 160.00 (143.50;179.50) | 8.06 |
|  | pCT T, µm | 186.91±75.41 | 179.64±55.11 | 176.00 (134.00;250.50) | 175.00 (133.00;238.50) | 3.97 |
|  | pCT N, µm | 194.09±68.62 | 184.09±44.29 | 208.00 (124.00;249.50) | 181.00 (151.00;219.00) | 5.29 |
| SSc - diffuse | mTCA, µm^2^ | 312 236.87±61 870.03 | 318 601.08±58 637.66 | 310 079.50 (271 055.50;338 186.00) | 313 904.75 (289 396.50;348 313.38) | -2.02 |
|  | mLA, µm^2^ | 211 641.45±45 660.25 | 214 672.00±39 193.94 | 214 406.00 (183 847.75;232 560.75) | 211 563.75 (195 026.88;243 728.38) | -1.42 |
|  | mSA, µm^2^ | 100 595.42±17 437.85 | 103 929.08±22 185.94 | 102 250.00 (88 452.75;106 712.25) | 99 109.25 (89 504.13;111 633.25) | -3.26 |
|  | mCVI, % | 67.51±2.49 | 67.40±2.75 | 67.84 (66.41;69.12) | 67.61 (66.72;69.39) | 0.17 |
|  | Central macular choroidal thickness, µm | 287.00±62.93 | 285.90±56.22 | 288.00 (259.50;303.50) | 283.00 (266.00;319.25) | 0.38 |
|  | SFCT, µm | 283.26±66.07 | 289.85±56.95 | 282.00 (244.00;313.00) | 275.50 (243.50;338.50) | -2.30 |
|  | Central macular choroidal volume, µm^3^ | 0.23±0.05 | 0.23±0.04 | 0.22 (0.21;0.24) | 0.22 (0.21;0.25) | 0.82 |
|  | Total choroidal volume, µm^3^ | 7.49±1.36 | 7.42±1.65 | 7.42 (6.65;7.99) | 7.31 (6.55;8.33) | 0.91 |
|  | pTCA, µm^2^ | 2 440 812.75±694 099.30 | 2 409 341.25±777 660.62 | 2 297 447.25 (2 016 699.38;3 052 401.00) | 2 256 859.25 (1 878 205.38;2 886 627.00) | 1.30 |
|  | pLA, µm^2^ | 1 577 700.57±463 263.03 | 1 544 736.13±526 051.28 | 1 457 415.25 (1 248 698.38;1 971 398.13) | 1 425 097.25 (1 171 398.38;1 975 565.50) | 2.11 |
|  | pSA, µm^2^ | 863 112.18±236 136.60 | 864 605.13±259 197.72 | 804 928.25 (713 778.75;1 035 451.50) | 799 725.00 (712 466.00;961 023.88) | -0.17 |
|  | pCVI, % | 64.49±1.73 | 63.74±2.38 | 64.52 (63.24;65.71) | 63.94 (62.19;65.08) | 1.17 |
|  | pCT Global, µm | 197.59±63.44 | 194.18±63.62 | 183.50 (158.00;242.00) | 175.50 (145.75;246.75) | 1.74 |
|  | pCT S, µm | 206.05±61.42 | 207.55±67.10 | 201.00 (173.75;253.00) | 191.50 (157.25;258.75) | -0.73 |
|  | pCT I, µm | 179.77±74.14 | 175.23±62.07 | 175.50 (118.50;228.25) | 157.50 (127.50;227.50) | 2.56 |
|  | pCT T, µm | 211.91±75.16 | 203.64±72.12 | 196.00 (164.75;260.00) | 185.50 (157.25;263.25) | 3.98 |
|  | pCT N, µm | 192.91±55.38 | 190.73±60.22 | 194.50 (171.00;228.50) | 177.00 (150.75;241.25) | 1.14 |

Abbreviations: CT, choroidal thickness; CVI, choroidal vascularity index; I, inferior; LA, luminal area; m, macular; N, nasal; p, peripapillary; Q1, quartile 1; Q3, quartile 3; S, superior; SA, stromal area; SD, standard deviation; SFCT, subfoveal choroidal thickness; T, temporal; TCA, total choroidal area

Relative mean difference calculated as per formula: (left eye - right eye) / average from both eyes * 100

**Supplementary Table 4.2.** Comparison of the choroidal parameters in fellow eyes in SSc patients by SSc subtype

| Group | Variable | ICC between both eyes | 95% CI for ICC | r between both eyes | p value for r | p-value of Wilcoxon test (w/o correction)^a^ | p-value of Wilcoxon test (B-H correction)^a^ |
| --- | --- | --- | --- | --- | --- | --- | --- |
| SSc - limited | mTCA | 0.790 | 0.357-0.948 | 0.77 | **0.021** | 0.359 | 0.479 |
|  | mLA | 0.785 | 0.344-0.946 | 0.72 | **0.037** | 0.301 | 0.479 |
|  | mSA | 0.791 | 0.359-0.948 | 0.77 | **0.021** | 0.496 | 0.567 |
|  | mCVI | 0.792 | 0.298-0.950 | 0.58 | 0.108 | >0.999 | >0.999 |
|  | Central macular choroidal thickness | 0.782 | 0.336-0.945 | 0.83 | **0.008** | 0.301 | 0.479 |
|  | SFCT | 0.728 | 0.224-0.930 | 0.83 | **0.008** | 0.203 | 0.479 |
|  | Central macular choroidal volume | 0.760 | 0.280-0.939 | 0.84 | **0.005** | 0.161 | 0.479 |
|  | Total choroidal volume | 0.896 | 0.602-0.976 | 0.93 | **<0.001** | 0.123 | 0.479 |
|  | pTCA | 0.917 | 0.630-0.980 | 0.95 | **<0.001** | 0.064 | 0.288 |
|  | pLA | 0.926 | 0.587-0.983 | 0.95 | **<0.001** | **0.037** | 0.288 |
|  | pSA | 0.883 | 0.611-0.969 | 0.94 | **<0.001** | 0.106 | 0.318 |
|  | pCVI | 0.631 | 0.088-0.891 | 0.43 | 0.218 | 0.322 | 0.450 |
|  | pCT Global | 0.915 | 0.730-0.976 | 0.92 | **<0.001** | 0.350 | 0.450 |
|  | pCT S | 0.962 | 0.871-0.990 | 0.93 | **<0.001** | 0.230 | 0.414 |
|  | pCT I | 0.787 | 0.406-0.937 | 0.83 | **0.003** | 0.168 | 0.378 |
|  | pCT T | 0.898 | 0.677-0.971 | 0.92 | **<0.001** | 0.563 | 0.563 |
|  | pCT N | 0.742 | 0.303-0.923 | 0.69 | **0.019** | 0.520 | 0.563 |
| SSc - diffuse | mTCA | 0.679 | 0.329-0.863 | 0.54 | **0.018** | 0.768 | >0.999 |
|  | mLA | 0.705 | 0.374-0.876 | 0.57 | **0.013** | 0.829 | >0.999 |
|  | mSA | 0.535 | 0.113-0.792 | 0.49 | **0.035** | 0.922 | >0.999 |
|  | mCVI | 0.434 | 0.001-0.739 | 0.47 | **0.044** | 0.922 | >0.999 |
|  | Central macular choroidal thickness | 0.689 | 0.335-0.872 | 0.61 | **0.007** | >0.999 | >0.999 |
|  | SFCT | 0.732 | 0.421-0.890 | 0.72 | **0.001** | 0.472 | >0.999 |
|  | Central macular choroidal volume | 0.676 | 0.311-0.866 | 0.63 | **0.005** | 0.962 | >0.999 |
|  | Total choroidal volume | 0.879 | 0.704-0.953 | 0.82 | **<0.001** | 0.887 | >0.999 |
|  | pTCA | 0.931 | 0.834-0.972 | 0.88 | **<0.001** | 0.841 | 0.898 |
|  | pLA | 0.93 | 0.833-0.972 | 0.88 | **<0.001** | 0.701 | 0.898 |
|  | pSA | 0.911 | 0.789-0.964 | 0.83 | **<0.001** | 0.898 | 0.898 |
|  | pCVI | 0.631 | 0.283-0.834 | 0.73 | **<0.001** | **0.033** | 0.297 |
|  | pCT Global | 0.938 | 0.858-0.974 | 0.92 | **<0.001** | 0.548 | 0.898 |
|  | pCT S | 0.886 | 0.745-0.951 | 0.80 | **<0.001** | 0.649 | 0.898 |
|  | pCT I | 0.896 | 0.768-0.955 | 0.91 | **<0.001** | 0.581 | 0.898 |
|  | pCT T | 0.936 | 0.852-0.973 | 0.92 | **<0.001** | 0.163 | 0.734 |
|  | pCT N | 0.873 | 0.719-0.945 | 0.90 | **<0.001** | 0.833 | 0.898 |

Abbreviations: CI, confidence interval; CT, choroidal thickness; CVI, choroidal vascularity index; I, inferior; ICC, intraclass correlation coefficient; LA, luminal area; m, macular; N, nasal; p, peripapillary; r, Spearman correlation coefficient; S, superior; SA, stromal area; SFCT, subfoveal choroidal thickness; T, temporal; TCA, total choroidal area

p<0.05 in bold font

Wilcoxon test comparing average level between left eyes and right eyes

a p-value after Benjamini-Hochberg correction for multiple comparisons. The correction was made separately for macular (8 comparisons) and peripapillary (9 comparisons) choroidal parameters.

**Supplementary Table 5.1.** Values of the choroidal parameters in SSc patients by Scl70 antibody presence

| Group | Variable | Mean ±SD left eyes | Mean ±SD right eyes | Median (Q1;Q3) left eyes | Median (Q1;Q3) right eyes | Relative mean difference (%) between left and right eyes |
| --- | --- | --- | --- | --- | --- | --- |
| SSc - Scl70 YES | mTCA, µm^2^ | 324 525.36±63 561.85 | 315 785.82±53 849.08 | 329 121.75 (292 859.63;349 168.25) | 308 837.25 (296 815.88;342 522.25) | 2.73 |
|  | mLA, µm^2^ | 220 921.14±46 405.55 | 213 994.79±35 548.05 | 223 458.00 (193 988.50;244 076.13) | 211 563.75 (205 058.75;237 048.75) | 3.19 |
|  | mSA, µm^2^ | 103 604.21±18 156.20 | 101 791.04±19 818.31 | 104 775.00 (98 871.13;106 884.63) | 99 109.25 (90 654.75;104 585.00) | 1.77 |
|  | mCVI, % | 67.86±2.23 | 67.82±1.77 | 68.06 (66.95;69.43) | 67.63 (66.87;69.23) | 0.07 |
|  | Central macular choroidal thickness, µm | 297.29±67.16 | 284.00±52.85 | 290.50 (262.00;352.75) | 283.00 (262.00;314.25) | 4.57 |
|  | SFCT, µm | 290.29±69.75 | 290.79±56.85 | 284.50 (242.50;340.50) | 293.00 (238.50;334.00) | -0.17 |
|  | Central macular choroidal volume, µm^3^ | 0.24±0.05 | 0.22±0.04 | 0.23 (0.21;0.28) | 0.23 (0.21;0.25) | 4.98 |
|  | Total choroidal volume, µm^3^ | 7.72±1.49 | 7.32±1.64 | 7.63 (6.90;8.76) | 7.35 (6.66;8.20) | 5.36 |
|  | pTCA, µm^2^ | 2 383 902.22±722 433.45 | 2 344 326.93±803 559.42 | 2 141 020.50 (2 004 297.25;3 134 333.00) | 2 256 859.25 (1 860 897.50;2 723 122.63) | 1.67 |
|  | pLA, µm^2^ | 1 539 767.78±476 845.57 | 1 493 694.11±531 575.50 | 1 392 568.75 (1 242 505.13;2 026 716.25) | 1 425 097.25 (1 164 354.00;1 758 073.75) | 3.04 |
|  | pSA, µm^2^ | 844 134.44±251 183.68 | 850 632.82±277 465.77 | 759 241.75 (700 582.00;1 047 846.38) | 783 562.75 (696 543.50;951 131.00) | -0.77 |
|  | pCVI, % | 64.49±1.73 | 63.40±2.14 | 64.52 (63.48;65.63) | 63.38 (62.31;64.99) | 1.70 |
|  | pCT Global, µm | 191.19±64.99 | 187.06±62.57 | 179.00 (152.25;244.50) | 172.50 (141.00;236.25) | 2.18 |
|  | pCT S, µm | 199.75±63.93 | 201.13±66.41 | 201.00 (160.50;251.25) | 186.00 (156.50;252.00) | -0.69 |
|  | pCT I, µm | 174.81±77.45 | 169.81±59.50 | 163.50 (110.00;232.50) | 157.50 (131.25;210.25) | 2.90 |
|  | pCT T, µm | 206.19±71.57 | 194.81±71.20 | 192.50 (168.25;254.25) | 185.50 (150.25;261.75) | 5.67 |
|  | pCT N, µm | 184.13±59.57 | 182.81±60.89 | 188.50 (144.00;225.50) | 170.00 (146.75;215.00) | 0.72 |
| SSc - Scl70 NO | mTCA, µm^2^ | 304 048.17±68 298.36 | 333 279.08±77 668.75 | 305 131.75 (264 314.75;341 989.25) | 336 537.00 (277 161.50;380 113.50) | -9.17 |
|  | mLA, µm^2^ | 203 269.88±46 016.50 | 222 175.31±48 116.47 | 205 537.50 (174 666.13;226 548.75) | 228 140.50 (192 296.50;253 930.00) | -8.89 |
|  | mSA, µm^2^ | 100 778.29±24 052.00 | 111 103.77±32 221.03 | 92 081.75 (88 724.88;121 714.25) | 113 542.00 (86 647.00;136 646.00) | -9.75 |
|  | mCVI, % | 66.84±2.77 | 66.96±3.79 | 66.51 (65.44;68.51) | 66.80 (63.51;70.73) | -0.18 |
|  | Central macular choroidal thickness, µm | 263.54±57.68 | 284.08±83.62 | 264.00 (232.00;288.00) | 272.00 (253.00;338.00) | -7.50 |
|  | SFCT, µm | 263.38±58.82 | 290.62±79.10 | 254.00 (227.00;282.00) | 270.00 (258.00;345.00) | -9.83 |
|  | Central macular choroidal volume, µm^3^ | 0.21±0.04 | 0.22±0.06 | 0.20 (0.18;0.23) | 0.21 (0.20;0.27) | -7.89 |
|  | Total choroidal volume, µm^3^ | 6.86±1.08 | 6.99±1.55 | 6.89 (5.99;7.37) | 7.15 (6.20;8.20) | -1.96 |
|  | pTCA, µm^2^ | 2 330 960.11±598 392.19 | 2 218 066.92±564 377.48 | 2 436 447.25 (1 884 602.63;2 793 838.13) | 2 007 611.00 (1 796 602.00;2 548 874.50) | 4.96 |
|  | pLA, µm^2^ | 1 508 623.46±410 465.23 | 1 421 834.77±386 637.06 | 1 553 999.00 (1 183 079.00;1 844 635.50) | 1 220 426.50 (1 161 232.50;1 689 500.00) | 5.92 |
|  | pSA, µm^2^ | 822 336.64±193 960.71 | 796 232.15±182 236.12 | 857 507.75 (686 692.88;947 740.63) | 787 184.50 (684 868.50;859 374.50) | 3.23 |
|  | pCVI, % | 64.54±1.84 | 63.87±1.88 | 64.20 (63.31;66.22) | 64.54 (61.99;65.15) | 1.04 |
|  | pCT Global, µm | 180.71±53.97 | 176.29±44.14 | 183.00 (133.25;231.75) | 171.50 (143.50;206.75) | 2.48 |
|  | pCT S, µm | 185.93±51.90 | 187.29±51.60 | 180.00 (140.50;226.75) | 177.50 (141.00;221.50) | -0.73 |
|  | pCT I, µm | 165.71±50.12 | 156.86±42.68 | 177.50 (119.50;206.50) | 153.50 (125.25;167.75) | 5.49 |
|  | pCT T, µm | 180.00±66.46 | 178.07±51.38 | 165.00 (137.25;227.25) | 167.00 (143.25;220.75) | 1.08 |
|  | pCT N, µm | 191.14±57.80 | 182.79±46.00 | 198.50 (140.25;231.25) | 174.00 (151.00;220.50) | 4.47 |

Abbreviations: CT, choroidal thickness; CVI, choroidal vascularity index; I, inferior; LA, luminal area; m, macular; N, nasal; p, peripapillary; Q1, quartile 1; Q3, quartile 3; S, superior; SA, stromal area; SD, standard deviation; SFCT, subfoveal choroidal thickness; T, temporal; TCA, total choroidal area

Relative mean difference calculated as per formula: (left eye - right eye) / average from both eyes * 100

**Supplementary Table 5.2.** Comparison of the choroidal parameters in fellow eyes in SSc patients by Scl70 antibody presence

| Group | Variable | ICC between both eyes | 95% CI for ICC | r between both eyes | p value for r | p-value of Wilcoxon test (w/o correction)^a^ | p-value of Wilcoxon test (B-H correction)^a^ |
| --- | --- | --- | --- | --- | --- | --- | --- |
| SSc - Scl70 YES | mTCA | 0.722 | 0.337-0.901 | 0.58 | **0.033** | 0.583 | 0.896 |
|  | mLA | 0.746 | 0.388-0.910 | 0.68 | **0.009** | 0.626 | 0.896 |
|  | mSA | 0.570 | 0.064-0.840 | 0.30 | 0.302 | 0.426 | 0.896 |
|  | mCVI | 0.218 | 0.001-0.668 | 0.45 | 0.112 | >0.999 | >0.999 |
|  | Central macular choroidal thickness | 0.699 | 0.251-0.899 | 0.74 | **0.004** | 0.685 | 0.896 |
|  | SFCT | 0.685 | 0.244-0.892 | 0.66 | **0.017** | 0.588 | 0.896 |
|  | Central macular choroidal volume | 0.698 | 0.248-0.898 | 0.72 | **0.005** | 0.783 | 0.896 |
|  | Total choroidal volume | 0.905 | 0.724-0.970 | 0.83 | **<0.001** | 0.784 | 0.896 |
|  | pTCA | 0.944 | 0.836-0.982 | 0.96 | **<0.001** | 0.808 | 0.980 |
|  | pLA | 0.940 | 0.828-0.980 | 0.92 | **<0.001** | 0.515 | 0.927 |
|  | pSA | 0.942 | 0.834-0.981 | 0.89 | **<0.001** | 0.903 | 0.980 |
|  | pCVI | 0.719 | 0.185-0.909 | 0.81 | **0.001** | 0.013 | 0.117 |
|  | pCT Global | 0.936 | 0.831-0.977 | 0.94 | **<0.001** | 0.393 | 0.927 |
|  | pCT S | 0.867 | 0.658-0.951 | 0.76 | **0.001** | 0.679 | 0.980 |
|  | pCT I | 0.887 | 0.711-0.959 | 0.91 | **<0.001** | 0.469 | 0.927 |
|  | pCT T | 0.924 | 0.793-0.973 | 0.93 | **<0.001** | 0.127 | 0.572 |
|  | pCT N | 0.881 | 0.693-0.957 | 0.87 | **<0.001** | 0.980 | 0.980 |
| SSc - Scl70 NO | mTCA | 0.794 | 0.406-0.937 | 0.80 | **0.003** | 0.092 | 0.516 |
|  | mLA | 0.789 | 0.400-0.935 | 0.73 | **0.009** | 0.129 | 0.516 |
|  | mSA | 0.766 | 0.383-0.926 | 0.75 | **0.007** | 0.204 | 0.544 |
|  | mCVI | 0.669 | 0.164-0.893 | 0.56 | 0.063 | 0.733 | 0.733 |
|  | Central macular choroidal thickness | 0.797 | 0.453-0.936 | 0.74 | **0.006** | 0.505 | 0.577 |
|  | SFCT | 0.753 | 0.366-0.921 | 0.71 | **0.009** | 0.289 | 0.577 |
|  | Central macular choroidal volume | 0.770 | 0.400-0.927 | 0.75 | **0.005** | 0.398 | 0.577 |
|  | Total choroidal volume | 0.869 | 0.605-0.961 | 0.91 | **<0.001** | 0.456 | 0.577 |
|  | pTCA | 0.884 | 0.674-0.963 | 0.90 | **<0.001** | 0.305 | 0.779 |
|  | pLA | 0.901 | 0.698-0.969 | 0.88 | **<0.001** | 0.110 | 0.495 |
|  | pSA | 0.822 | 0.518-0.942 | 0.86 | **<0.001** | 0.455 | 0.795 |
|  | pCVI | 0.667 | 0.207-0.885 | 0.62 | **0.029** | 0.068 | 0.495 |
|  | pCT Global | 0.903 | 0.733-0.968 | 0.90 | **<0.001** | 0.530 | 0.795 |
|  | pCT S | 0.947 | 0.844-0.983 | 0.93 | **<0.001** | 0.730 | 0.821 |
|  | pCT I | 0.831 | 0.567-0.942 | 0.85 | **<0.001** | 0.346 | 0.779 |
|  | pCT T | 0.894 | 0.703-0.965 | 0.90 | **<0.001** | >0.999 | >0.999 |
|  | pCT N | 0.748 | 0.391-0.911 | 0.81 | **<0.001** | 0.670 | 0.821 |

Abbreviations: CI, confidence interval; CT, choroidal thickness; CVI, choroidal vascularity index; I, inferior; ICC, intraclass correlation coefficient; LA, luminal area; m, macular; N, nasal; p, peripapillary; r, Spearman correlation coefficient; S, superior; SA, stromal area; SFCT, subfoveal choroidal thickness; T, temporal; TCA, total choroidal area

p<0.05 in bold font

Wilcoxon test comparing average level between left eyes and right eyes

a p-value after Benjamini-Hochberg correction for multiple comparisons. The correction was made separately for macular (8 comparisons) and peripapillary (9 comparisons) choroidal parameters.

**Supplementary Table 6.1.** Values of the choroidal parameters in SSc patients by digital ulcers presence

| Group | Variable | Mean ±SD left eyes | Mean ±SD right eyes | Median (Q1;Q3) left eyes | Median (Q1;Q3) right eyes | Relative mean difference (%) between left and right eyes |
| --- | --- | --- | --- | --- | --- | --- |
| SSc - digital ulcers present/ in history - NO | mTCA, µm^2^ | 307 456.63±56 056.97 | 321 452.19±64 426.74 | 309 559.75 (268 350.00;335 104.63) | 309 306.50 (277 161.50;347 737.50) | -4.45 |
|  | mLA, µm^2^ | 205 988.58±39 912.16 | 216 233.10±41 235.68 | 208 590.25 (182 176.88;228 638.00) | 213 933.50 (192 296.50;240 463.00) | -4.85 |
|  | mSA, µm^2^ | 101 468.05±17 933.93 | 105 219.10±24 980.26 | 101 481.25 (90 308.38;107 025.75) | 97 681.50 (86 647.00;113 542.00) | -3.63 |
|  | mCVI, % | 66.82±2.51 | 67.36±2.44 | 66.75 (65.98;68.03) | 67.41 (66.56;69.14) | -0.81 |
|  | Central macular choroidal thickness, µm | 275.95±57.65 | 281.48±70.71 | 283.50 (235.75;301.75) | 272.00 (253.00;312.00) | -1.98 |
|  | SFCT, µm | 271.85±61.40 | 287.67±69.50 | 278.00 (230.00;296.25) | 274.00 (236.00;338.00) | -5.65 |
|  | Central macular choroidal volume, µm^3^ | 0.22±0.04 | 0.22±0.05 | 0.22 (0.18;0.24) | 0.21 (0.20;0.25) | -2.25 |
|  | Total choroidal volume, µm^3^ | 7.12±1.19 | 6.97±1.56 | 7.19 (6.12;7.63) | 7.15 (6.20;7.87) | 2.09 |
|  | pTCA, µm^2^ | 2 242 945.80±599 527.56 | 2 148 913.80±559 593.59 | 2 077 751.25 (1 889 141.88;2 658 443.63) | 2 007 160.75 (1 789 105.50;2 455 774.38) | 4.28 |
|  | pLA, µm^2^ | 1 451 179.98±411 841.33 | 1 369 127.25±382 135.91 | 1 353 485.00 (1 223 464.50;1 706 861.00) | 1 251 218.00 (1 136 835.75;1 618 834.25) | 5.82 |
|  | pSA, µm^2^ | 791 765.82±193 809.95 | 779 786.55±182 698.87 | 759 241.75 (682 295.13;937 615.13) | 750 380.50 (674 422.63;882 591.38) | 1.52 |
|  | pCVI, % | 64.48±1.83 | 63.41±2.03 | 64.38 (63.09;65.81) | 63.60 (61.75;65.06) | 1.66 |
|  | pCT Global, µm | 175.68±55.84 | 168.36±44.29 | 176.00 (138.75;207.50) | 155.00 (139.75;189.75) | 4.25 |
|  | pCT S, µm | 184.91±54.78 | 179.86±48.77 | 182.50 (147.00;222.75) | 167.50 (152.25;209.50) | 2.77 |
|  | pCT I, µm | 157.18±58.80 | 150.18±42.57 | 148.00 (114.00;190.25) | 146.00 (127.50;161.00) | 4.55 |
|  | pCT T, µm | 183.14±66.09 | 172.55±53.29 | 171.50 (137.25;211.25) | 163.00 (139.00;186.25) | 5.96 |
|  | pCT N, µm | 177.45±57.64 | 171.14±45.91 | 184.00 (129.00;224.00) | 159.50 (143.25;207.25) | 3.62 |
| SSc - digital ulcers present/ in history - YES | mTCA, µm^2^ | 340 258.94±77 497.69 | 346 977.72±68 380.39 | 344 161.50 (314 941.13;381 889.88) | 350 041.00 (312 147.00;397 890.50) | -1.96 |
|  | mLA, µm^2^ | 232 709.56±52 680.65 | 230 888.78±41 668.26 | 235 280.75 (207 129.50;267 443.88) | 245 606.00 (203 059.50;264 974.00) | 0.79 |
|  | mSA, µm^2^ | 107 549.38±25 849.55 | 116 088.94±29 687.29 | 110 751.50 (98 664.63;120 786.88) | 122 566.00 (104 435.00;140 559.50) | -7.64 |
|  | mCVI, % | 68.50±1.90 | 66.91±3.76 | 68.29 (67.64;69.99) | 66.86 (64.10;69.79) | 2.35 |
|  | Central macular choroidal thickness, µm | 293.78±71.78 | 315.22±59.96 | 284.00 (259.00;368.00) | 332.00 (284.00;364.00) | -7.04 |
|  | SFCT, µm | 298.33±67.77 | 320.33±57.26 | 308.00 (254.00;354.00) | 340.00 (309.00;358.00) | -7.11 |
|  | Central macular choroidal volume, µm^3^ | 0.23±0.06 | 0.25±0.05 | 0.22 (0.20;0.29) | 0.26 (0.22;0.28) | -6.48 |
|  | Total choroidal volume, µm^3^ | 7.82±1.51 | 8.27±1.55 | 7.65 (7.42;8.90) | 8.31 (7.48;8.78) | -5.66 |
|  | pTCA, µm^2^ | 2 832 268.68±698 474.31 | 2 792 741.30±797 906.49 | 3 130 353.00 (2 381 223.75;3 325 760.25) | 2 829 292.25 (2 363 751.88;3 424 002.75) | 1.41 |
|  | pLA, µm^2^ | 1 830 645.59±454 568.56 | 1 817 935.90±529 005.84 | 2 002 402.00 (1 546 804.00;2 143 723.00) | 1 888 639.50 (1 516 242.63;2 280 451.75) | 0.70 |
|  | pSA, µm^2^ | 1 001 623.09±247 717.32 | 974 805.40±278 470.51 | 1 046 614.00 (848 166.50;1 182 037.25) | 932 134.75 (832 353.13;1 153 773.88) | 2.71 |
|  | pCVI, % | 64.66±1.40 | 64.98±2.18 | 64.48 (64.18;65.56) | 64.76 (63.93;66.04) | -0.48 |
|  | pCT Global, µm | 229.36±61.11 | 231.00±59.85 | 242.00 (201.50;265.00) | 236.00 (197.50;281.00) | -0.71 |
|  | pCT S, µm | 229.36±61.85 | 248.64±64.79 | 250.00 (192.50;263.50) | 255.00 (201.50;297.50) | -8.06 |
|  | pCT I, µm | 218.91±67.07 | 210.36±56.37 | 226.00 (195.50;262.50) | 214.00 (191.00;255.50) | 3.98 |
|  | pCT T, µm | 244.45±77.97 | 241.82±70.00 | 251.00 (209.00;280.00) | 253.00 (201.00;282.00) | 1.08 |
|  | pCT N, µm | 225.00±50.14 | 223.27±56.47 | 224.00 (202.50;258.50) | 236.00 (188.50;264.50) | 0.77 |

Abbreviations: CT, choroidal thickness; CVI, choroidal vascularity index; I, inferior; LA, luminal area; m, macular; N, nasal; p, peripapillary; Q1, quartile 1; Q3, quartile 3; S, superior; SA, stromal area; SD, standard deviation; SFCT, subfoveal choroidal thickness; T, temporal; TCA, total choroidal area

Relative mean difference calculated as per formula: (left eye - right eye) / average from both eyes * 100

**Supplementary Table 6.2.** Comparison of the choroidal parameters in fellow eyes in SSc patients by digital ulcers presence

| Group | Variable | ICC between both eyes | 95% CI for ICC | r between both eyes | p value for r | p-value of Wilcoxon test (w/o correction)^a^ | p-value of Wilcoxon test (B-H correction)^a^ |
| --- | --- | --- | --- | --- | --- | --- | --- |
| SSc - digital ulcers present/ in history - NO | mTCA | 0.743 | 0.465-0.889 | 0.61 | **0.005** | 0.294 | 0.698 |
|  | mLA | 0.726 | 0.437-0.881 | 0.55 | **0.014** | 0.231 | 0.698 |
|  | mSA | 0.754 | 0.481-0.894 | 0.73 | **<0.001** | 0.812 | 0.984 |
|  | mCVI | 0.676 | 0.354-0.856 | 0.69 | 0.001 | 0.349 | 0.698 |
|  | Central macular choroidal thickness | 0.784 | 0.520-0.911 | 0.76 | **<0.001** | 0.984 | 0.984 |
|  | SFCT | 0.762 | 0.490-0.900 | 0.74 | **<0.001** | 0.286 | 0.698 |
|  | Central macular choroidal volume | 0.760 | 0.477-0.900 | 0.76 | **<0.001** | 0.887 | 0.984 |
|  | Total choroidal volume | 0.876 | 0.712-0.950 | 0.88 | **<0.001** | 0.862 | 0.984 |
|  | pTCA | 0.891 | 0.747-0.955 | 0.87 | **<0.001** | 0.165 | 0.297 |
|  | pLA | 0.899 | 0.740-0.960 | 0.88 | **<0.001** | 0.053 | 0.234 |
|  | pSA | 0.851 | 0.663-0.938 | 0.82 | **<0.001** | 0.388 | 0.499 |
|  | pCVI | 0.665 | 0.161-0.871 | 0.71 | **0.001** | **0.003** | **0.027** |
|  | pCT Global | 0.881 | 0.738-0.949 | 0.91 | **<0.001** | 0.104 | 0.234 |
|  | pCT S | 0.870 | 0.717-0.944 | 0.84 | **<0.001** | 0.581 | 0.581 |
|  | pCT I | 0.802 | 0.586-0.912 | 0.85 | **<0.001** | 0.306 | 0.459 |
|  | pCT T | 0.876 | 0.723-0.947 | 0.88 | **<0.001** | 0.088 | 0.234 |
|  | pCT N | 0.763 | 0.515-0.894 | 0.78 | **<0.001** | 0.463 | 0.521 |
| SSc - digital ulcers present/ in history - YES | mTCA | 0.686 | 0.001-0.930 | 0.36 | 0.389 | 0.945 | 0.945 |
|  | mLA | 0.730 | 0.110-0.940 | 0.62 | 0.115 | 0.945 | 0.945 |
|  | mSA | 0.559 | 0.001-0.893 | 0.19 | 0.665 | 0.547 | 0.729 |
|  | mCVI | 0.429 | 0.001-0.843 | 0.55 | 0.171 | 0.313 | 0.501 |
|  | Central macular choroidal thickness | 0.603 | 0.001-0.903 | 0.55 | 0.171 | 0.313 | 0.501 |
|  | SFCT | 0.615 | 0.001-0.906 | 0.52 | 0.197 | 0.313 | 0.501 |
|  | Central macular choroidal volume | 0.587 | 0.001-0.898 | 0.56 | 0.146 | 0.313 | 0.501 |
|  | Total choroidal volume | 0.887 | 0.545-0.976 | 0.64 | 0.096 | 0.161 | 0.501 |
|  | pTCA | 0.939 | 0.774-0.985 | 0.92 | **<0.001** | 0.922 | >0.999 |
|  | pLA | 0.937 | 0.766-0.984 | 0.89 | **0.001** | >0.999 | >0.999 |
|  | pSA | 0.922 | 0.720-0.980 | 0.94 | **<0.001** | 0.625 | >0.999 |
|  | pCVI | 0.532 | 0.001-0.861 | 0.54 | 0.113 | 0.695 | >0.999 |
|  | pCT Global | 0.962 | 0.876-0.990 | 0.93 | **<0.001** | 0.689 | >0.999 |
|  | pCT S | 0.912 | 0.447-0.980 | 0.96 | **<0.001** | **0.004** | **0.036** |
|  | pCT I | 0.895 | 0.674-0.970 | 0.81 | **0.004** | 0.374 | >0.999 |
|  | pCT T | 0.956 | 0.846-0.988 | 0.92 | **<0.001** | 0.722 | >0.999 |
|  | pCT N | 0.851 | 0.543-0.958 | 0.79 | **0.006** | 0.898 | >0.999 |

Abbreviations: CI, confidence interval; CT, choroidal thickness; CVI, choroidal vascularity index; I, inferior; ICC, intraclass correlation coefficient; LA, luminal area; m, macular; N, nasal; p, peripapillary; r, Spearman correlation coefficient; S, superior; SA, stromal area; SFCT, subfoveal choroidal thickness; T, temporal; TCA, total choroidal area

p<0.05 in bold font

Wilcoxon test comparing average level between left eyes and right eyes

a p-value after Benjamini-Hochberg correction for multiple comparisons. The correction was made separately for macular (8 comparisons) and peripapillary (9 comparisons) choroidal parameters.
